# Environmental changes affect the microbial release of hydrogen sulfide and methane from sediments at Boknis Eck (SW Baltic Sea)

**DOI:** 10.3389/fmicb.2022.1096062

**Published:** 2022-12-21

**Authors:** Mirjam Perner, Klaus Wallmann, Nicole Adam-Beyer, Helmke Hepach, Katja Laufer-Meiser, Stefanie Böhnke, Isabel Diercks, Hermann W. Bange, Daniela Indenbirken, Verena Nikeleit, Casey Bryce, Andreas Kappler, Anja Engel, Florian Scholz

**Affiliations:** ^1^Department of Marine Biogeochemistry, GEOMAR Helmholtz Centre for Ocean Research, Kiel, Germany; ^2^Leibniz Institute of Virology, Hamburg, Germany; ^3^Department of Geomicrobiology and Geosciences, University of Tübingen, Tübingen, Germany; ^4^School of Earth Sciences, University of Bristol, Bristol, United Kingdom

**Keywords:** microbial sulfide oxidation, anaerobic oxidation of methane, hypoxia, marine sediments, sulfate reduction rates, sedimentary microbial community

## Abstract

Anthropogenic activities are modifying the oceanic environment rapidly and are causing ocean warming and deoxygenation, affecting biodiversity, productivity, and biogeochemical cycling. In coastal sediments, anaerobic organic matter degradation essentially fuels the production of hydrogen sulfide and methane. The release of these compounds from sediments is detrimental for the (local) environment and entails socio-economic consequences. Therefore, it is vital to understand which microbes catalyze the re-oxidation of these compounds under environmental dynamics, thereby mitigating their release to the water column. Here we use the seasonally dynamic Boknis Eck study site (SW Baltic Sea), where bottom waters annually fall hypoxic or anoxic after the summer months, to extrapolate how the microbial community and its activity reflects rising temperatures and deoxygenation. During October 2018, hallmarked by warmer bottom water and following a hypoxic event, modeled sulfide and methane production and consumption rates are higher than in March at lower temperatures and under fully oxic bottom water conditions. The microbial populations catalyzing sulfide and methane metabolisms are found in shallower sediment zones in October 2018 than in March 2019. DNA-and RNA profiling of sediments indicate a shift from primarily organotrophic to (autotrophic) sulfide oxidizing Bacteria, respectively. Previous studies using data collected over decades demonstrate rising temperatures, decreasing eutrophication, lower primary production and thus less fresh organic matter transported to the Boknis Eck sediments. Elevated temperatures are known to stimulate methanogenesis, anaerobic oxidation of methane, sulfate reduction and essentially microbial sulfide consumption, likely explaining the shift to a phylogenetically more diverse sulfide oxidizing community based on RNA.

## Introduction

Coastal marine environments play a pivotal role for element transformations and local economy. They are, however, very susceptible to anthropogenic activities. Human-triggered changes include increasing atmospheric carbon dioxide mole fractions accompanied by rising temperatures, ocean acidification (increasing carbon dioxide levels) and ocean deoxygenation (declining oxygen concentrations; Schmidtko et al., 2017; Brauko et al., 2020). Ongoing deoxygenation has promoted the expansion of hypoxia (<62.5 μM oxygen) and anoxia, affecting oceanic biodiversity, productivity and biogeochemical cycling (Breitburg et al., 2018; Brauko et al., 2020).

Marine sediments cover ~70% of Earth’s surface and are the largest long-term carbon sink on Earth (LaRowe et al., 2020). Theoretically, microbial degradation of sedimentary organic matter (OM) occurs along a vertical redox cascade, with oxygen as an electron acceptor in the oxic layer followed by alternative electron acceptors, i.e., nitrate, metal oxides and sulfate, as well as fermentative processes and methanogenesis (Middelburg, 2018); although different redox reactions appear to occur in parallel, if sufficient OM is available (e.g., Sela-Adler et al., 2017). Depending on the microbial metabolism, a variety of reduced metabolic products are released from the microbial cells: hydrogen sulfide is biologically produced if OM degradation or anaerobic oxidation of methane (AOM) are coupled to sulfate reduction (SR) while methane is generated by methanogens in the final step of sedimentary OM degradation (Middelburg, 2018). These reduced solutes diffuse upwards in the sediment column until they come into contact with available electron acceptors and are oxidized. Although microbes can generate energy by exploiting this chemical disequilibrium, a fraction of these reduced species may not be oxidized and emitted into the overlying bottom waters. Some of these products like hydrogen sulfide and the greenhouse gas methane have negative impacts on the function of immediate marine ecosystems and beyond. For example, hydrogen sulfide is toxic and can cause marine species’ mortality, affecting the food webs (Vaquer-Sunyer and Duarte, 2008) and posing a threat to fishery-based economics (Diaz and Rosenberg, 2008; Levin et al., 2009). Recent modeling studies indicate that the benthic release of toxic sulfide may be intensified by ongoing ocean warming and deoxygenation (Wallmann et al., 2022b).

The shallow (28 m) Boknis Eck time-series station^1^ in the Eckernförder Bay (SW Baltic Sea) is a well-known site to study the benthic-pelagic coupling of nutrient cycles and redox processes. The OM contents of Boknis Eck sediments are overall high and phytoplankton blooms in spring, summer and autumn provide high OM fluxes to the seafloor (Smetacek et al., 1984; Smetacek, 1985). Enhanced OM degradation leads to elevated respiration rates, increased oxygen demand, consolidating developed hypoxia or even anoxia during certain times of the year (Lennartz et al., 2014). Sluggish deep-water renewal and restricted seafloor topography amplify local oxygen depletion (Dietze and Löptien, 2021). High benthic degradation rates under anoxic conditions support increased rates of SR and methanogenesis producing hydrogen sulfide and methane, respectively (Treude et al., 2005; Bertics et al., 2013; Maltby et al., 2018). However, over the last decades Boknis Eck has been exposed to environmental change and experienced a clear shift towards warmer water temperatures, higher frequencies of temperature anomalies, and an ongoing decline in bottom water oxygen levels, despite decreasing eutrophication and primary production (Lennartz et al., 2014). We here compare and discuss geochemical and microbiological data from Boknis Eck sediments exposed to warmer, previously hypoxic bottom waters (October 2018) with those subjected to colder, fully oxic bottom waters (March 2019). We aim at understanding how environmental changes, in particular temperature and related deoxygenation, affect the microbial community, as reflected by DNA-and RNA-profiling, and their buffer ability to mitigate hydrogen sulfide and methane emissions from sediments.

## Materials and methods

Discrete seawater samples for the determination of dissolved nutrients (nitrate, nitrite, ammonium, phosphate and silicate), oxygen and methane concentrations were taken on a monthly basis between January 2018 and April 2019 as part of the regular sampling program at Boknis Eck (Eckernförde Bay, SW Baltic Sea, Germany) (54°31.20’N, 10°02.50′E). Furthermore, water column samples were taken for the analysis of components of dissolved organic matter (DOM) including dissolved amino acids and dissolved organic carbon (DOC), as well as nano-and pico-plankton cell counts as part of the regular sampling program in March and October from 2015 to 2019 with DOC measurements starting only in October of 2016 (see Piontek et al., 2022 for analyses). Nano- (2–20 μM) and pico-plankton (< 2 μM) cell counts were divided into two groups based on the pigments chlorophyll *a* (Chl *a*) and phycoerythrin (phy). The latter group includes small cyanobacteria such as *Synechococcus*. DOM and phytoplankton cell counts were integrated over the whole water column between the depths 1 m and 25 m. Additionally, in two sampling campaigns in autumn (23rd October) 2018 and spring (15th March) 2019, a total of seven sediment cores were collected at Boknis Eck using a minicorer (MIC) device. Hydrogen sulfide, sulfate, manganese and iron (Fe) concentrations, total alkalinity and chloride were determined for the sediment pore waters. Fe oxide and sulfide minerals were analyzed for the sediments. A non-steady state model was set up to evaluate pore water data and quantify metabolic rates for Boknis Eck sediments. Microbiological analyses were conducted for the sediment layers down to a maximum of 26 cm. They included bacterial and archaeal 16S tag sequencing of DNA and RNA (cDNA), cell number enumeration, CARD-FISH (catalyzed-reported deposition-fluorescence *in situ* hybridization) and qPCR (quantitative PCR). An overview of the analyses conducted for the different sediment cores is given in Supplementary Table S1. All procedures for geochemical analyses, the reactive transport model and microbiological analyses are detailed in the Supplementary material and methods S1–S4.

## Results and discussion

**Figure 1:**
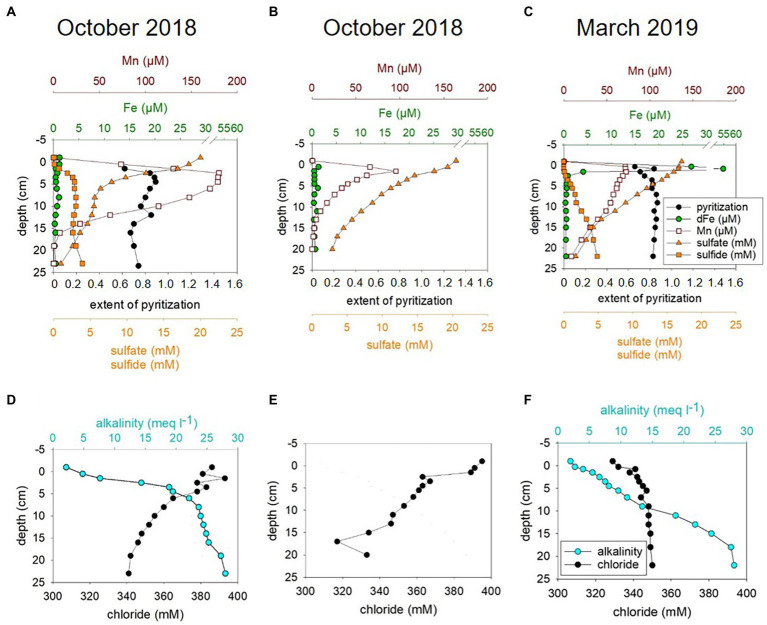
Pore water chemistry for MIC1 (Deployment, D1) for October 2018 **(A,D)**, MIC2 (D2) for October 2018 **(B,E)**, and for MIC1 (D1) for March 2019 **(C,F)**.

**Figure 2:**
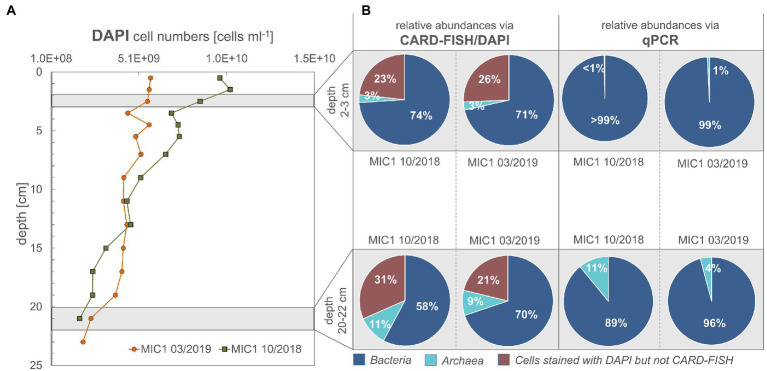
**(A,B)** Cell numbers for sediment depths according to DAPI staining, CARD-FISH and qPCR data. Cells counted are the sum of cells from pore water and those detached from sediment particles by ultrasonication.

**Figure 3:**
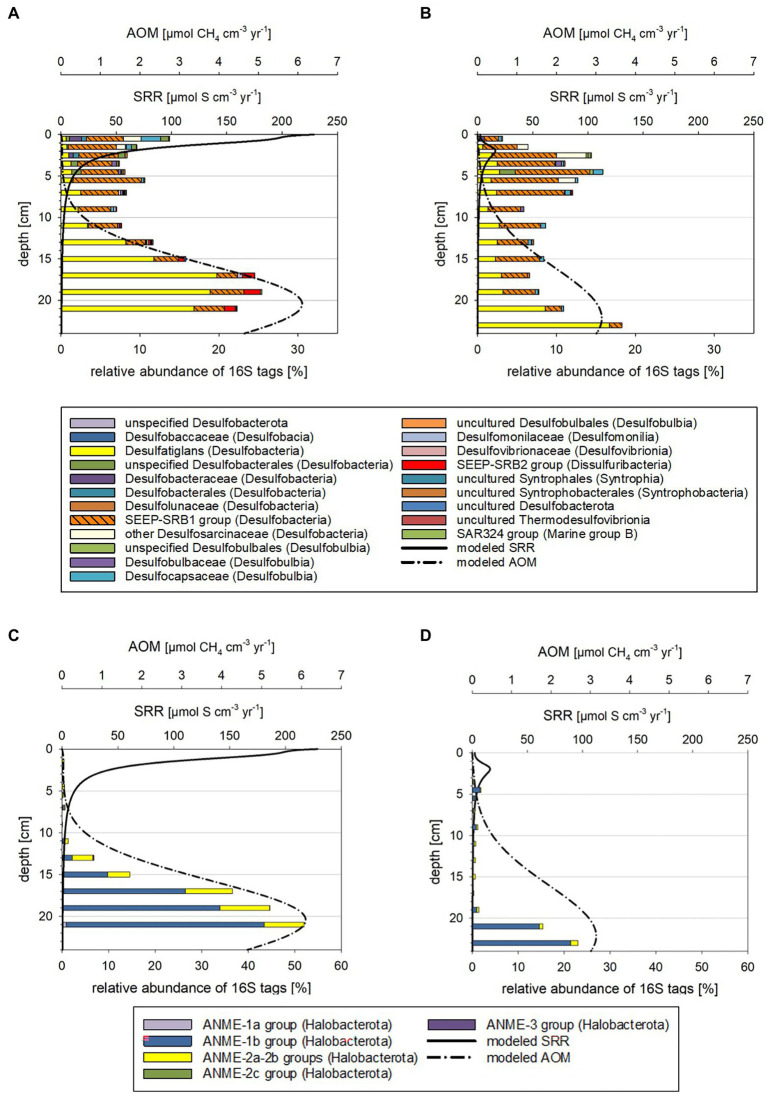
Taxonomy plots of RNA-based 16S tags of potential sulfate-reducing Bacteria **(A,B)** and anaerobic methane oxidizers **(C,D)** alongside modeled rates of SR and AOM for October 2018 **(A,C)** and March 2019 **(B,D)**.

**Figure 4:**
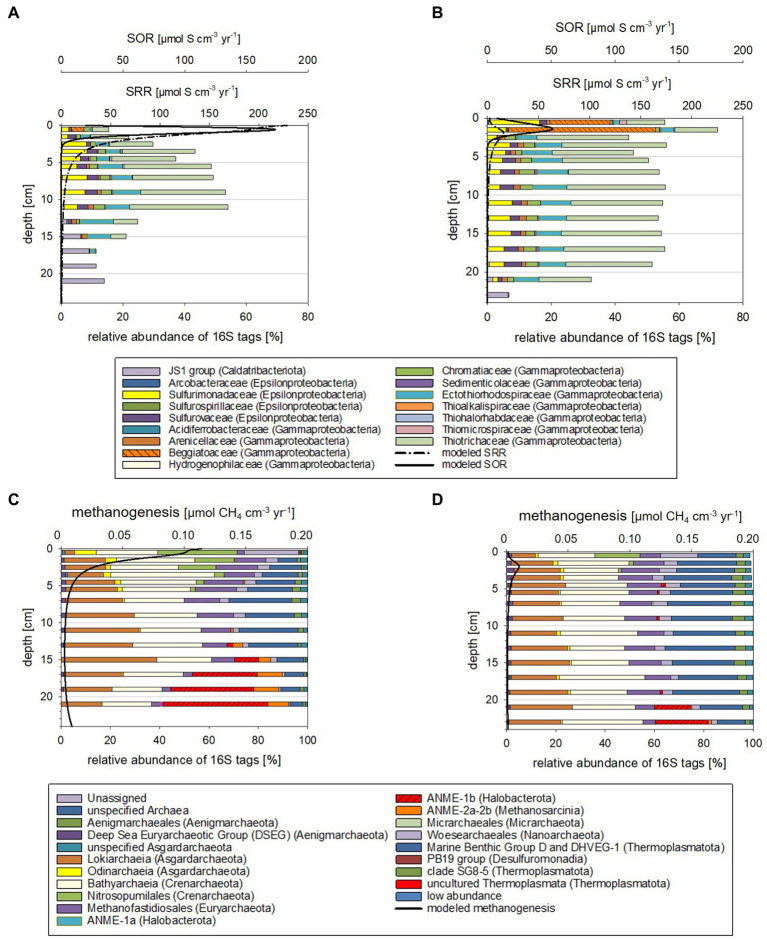
Taxonomy plots of RNA-based 16S tags of Bacteria potentially oxidizing reduced sulfur compounds **(A,B)** and Archaea **(C,D)** alongside modeled rates for SOR and methanogenesis for October 2018 **(A,C)** and March 2019 **(B,D)**.

**Figure 5:**
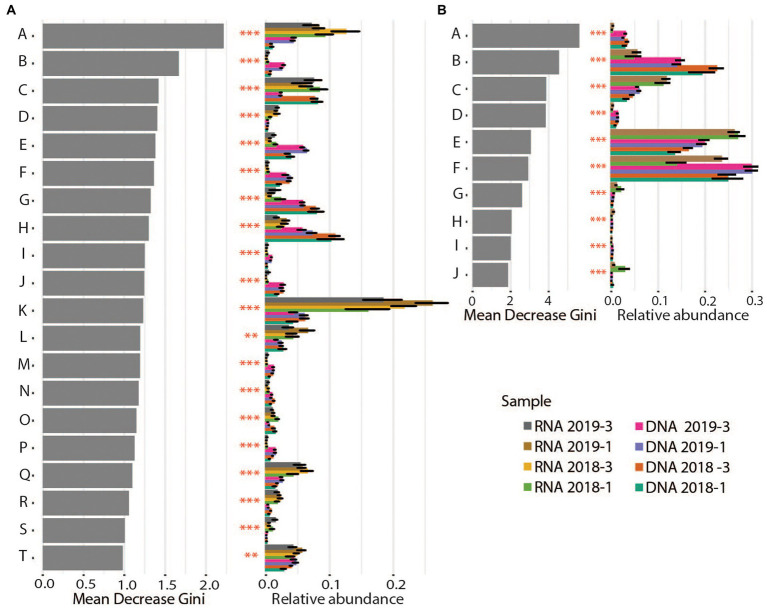
Differential abundance analyses for Bacteria **(A)** and Archaea **(B)** on family and order level, respectively. Gray bars show the value for “Mean decrease Gini,” which is a measure for the importance of the taxon for the difference between the samples. Colored bars show the relative abundance per sediment core (averages and standard deviation) for the different taxonomic groups. Stars illustrate the significance of the difference between cores. Bacteria: A: *Pirellulaceae*, B: Latescibacterota, C: Bacteroidetes_BD2–2, D: *Arenicellaceae*, E: *Anaerolineaceae*, F: *Sandaracinaceae*, G: *Flavobacteriaceae*, H: *Sulfurovaceae*, I: OM190, J: *Woeseiaceae*, K: *Thiotrichaceae*, L: *Sulfurimonadaceae*, M: *Calditrichaceae*, N: Sva0485, O: SB − 5, P: *Desulfobulbaceae*, Q: *Ectothiorhodospiraceae*, R: *Methylomonadaceae*, S: *Clostridiaceae*, T: B2M28. Archaea: A: Micrarchaeales, B: Woesearchaeales, C: Methanofastidiosales, D: Aenigmarchaeales, E: Bathyarchaeia, F: Marine Benthic Group D and DHVEG−1, G: Odinarchaeia, H: Thermoplasmatota, I: Iainarchaeales, J: Methanosarcinales.

In the current study we investigated how the sedimentary microbial community and its catalytic ability to buffer sulfide and methane release from the sediments is affected by ongoing environmental modifications. For studying seasonal effects, we used material collected in October 2018 and March 2019 and included numerous environmental factors into our analyses. This included water chemistry (Supplementary Figure S1) and phytoplankton data as well as total amino acid concentrations (Supplementary Figure S2) from the water column (surface to bottom waters at 25 m). Sediments were investigated down to 26 cm sediment depth and analyses combined pore water chemistry (Figure 1), solid phase reactive iron species (Supplementary Figure S3), cell numbers and relative proportions of active sedimentary microbes (Figure 2). Modeled metabolic rates for sulfate reduction (SRR), AOM, sulfide oxidation (SOR) and methanogenesis (Figures 3, 4; Supplementary Figures S4, S5) were linked to 16S tags of bacterial and archaeal transcripts (Supplementary Figures S6, S7). Additionally, we compared microbial RNA-profiling data, considered to include currently active communities, with DNA-profiling expected to reflect communities adapted to current and previous environmental conditions (Figure 5).

### General characteristics at Boknis Eck

#### Environmental features of the water column

Our water column data for 2018 and 2019 (Supplementary Figure S1) agreed with the typical seasonal variability of water temperature, salinity, nutrient concentrations, and methane levels as also observed between 1957 and 2013 (Lennartz et al., 2014; Ma et al., 2020; Gindorf et al., 2022). Just before our sediment sampling campaign in October 2018, a hypoxic event started to develop in 25 m water depth (67 μM oxygen in August 2018) and reached minimum (i.e., suboxic) oxygen concentrations (6 μM) by mid-September (Supplementary Figure S1). Then, oxygen concentrations in 25 m water depth increased to 194 μM by mid-October 2018 (Supplementary Figure S1) indicating that the deep water at Boknis Eck had returned to oxic conditions. In March 2019 near bottom water oxygen concentrations in 25 m water depth were 276 μM (Supplementary Figure S1).

Nano-and pico-plankton cell counts (Supplementary Figures S2A,B) were in line with recent work, which has demonstrated an intensification of cyanobacteria blooms over the last decades (Beltran-Perez and Waniek, 2022). The shift in the composition of phytoplankton can lead to a difference in the composition of the particulate organic matter (POM) reaching the seafloor, but we currently have no data from the seafloor detailing the POM composition. The concentration of total dissolved amino acids (TDAA) in the water column, here used as a tracer for degraded OM (Gaye et al., 2022) reached maximum concentrations in October 2018 (Supplementary Figure S2C), coinciding with maximum picoplankton cell numbers (Supplementary Figure S2B). The higher microbial OM turnover in summer matched data from previous field and laboratory studies positing a temperature dependence of the carbon flux from the autotrophic into the heterotrophic community in the Baltic Sea (von Scheibner et al., 2018; Bunse et al., 2019). How these shifts in the phytoplankton community affect the POM rain rate and which proportion and quality of the OM actually reaches the sediments remains unclear. However, it is generally assumed that the highest particulate organic carbon (POC)-flux is generated in February/March with the onset of maximum productivity (Wasmund, 2017; Wallmann et al., 2022a). For more discussions on water column properties see Supplementary Material.

#### Geochemistry of the pore water/sediments

The geochemical data obtained for pore waters of sediments from October 2018 and March 2019 included hydrogen sulfide, sulfate, iron (Fe), manganese, alkalinity, and chloride. These data are summarized in Figure 1 and are discussed in more detail together with sedimentary reactive Fe (Supplementary Figure S3) and below under modeled metabolic rates.

In summary, the pore water profiles obtained in October 2018 were overall similar to those observed in March 2019. However, hydrogen sulfide concentrations above 12 cm were generally lower in March 2019 than in October 2018 and the surface sediment (0–1 cm) was non-sulfidic and characterized by a peak in dissolved Fe in March 2019 (Figures 1A–C; Supplementary results and discussion). Thus, redox conditions in surface sediments were less reducing in March 2019 which is in accordance with higher bottom water oxygen concentrations (Supplementary Figure S1). Less reducing conditions in March 2019 are also reflected in slightly lower abundances of culturable Fe(III)-reducing microorganisms that degrade OM or oxidize H_2_ in the sediments in March than in October (see Supplementary Figure S8; Supplementary results and discussion). In contrast to previous years, where hydrogen sulfide enrichment (>1 mM) did not start until about 10 cm sediment depth (Preisler et al., 2007; Dale et al., 2013), hydrogen sulfide already accumulated considerably (>1 mM) in the uppermost 2.5 cm in October 2018 and 5.5 cm in March 2019 (Figures 3A,C) suggestive of a higher sulfide flux relative to the years before. The alkalinity gradient in surface sediments was highest in October 2018 (Figures 1D,F) and may point to elevated SRR and AOM, since these processes produce considerable amounts of alkalinity (Meister et al., 2022 and references therein). More so, the alkalinity gradient particularly for 2018 but also 2019 was considerably steeper in shallower regions than in sediments monitored in 2010 (Dale et al., 2013).

#### Modeled sedimentary metabolic rates

A sediment transport-reaction model was applied to relate the observed seasonal variability to changing boundary conditions (i.e., bottom water oxygen, temperature and salinity, Supplementary Figure S1) and to derive microbial turnover rates of carbon and sulfur. Generally, the model achieved a good fit for October 2018 and March 2019 (Supplementary Figures S9, S10). However, in individual cores taken in October 2018 chloride depletion, dissolved sulfate and sulfide concentrations were not reproduced by the model (Figures 1D–F, Supplementary Figure S9) suggesting a strong small-scale spatial variability and rapid microbial processes restoring the diffusion-controlled sulfate profile. Possibly, the core was affected by deep-reaching bio-irrigation, since previous bromide tracer studies with Boknis Eck cores revealed that bottom water may be pumped down to sediment depths of up to 15 cm by benthic biota or other transport processes such as gas bubble ascent (Dale et al., 2013). These additional transport processes provide an episodic supply of oxygen and sulfate for deep sediment layers that are usually not exposed to these electron acceptors. They probably induce microbial oxidation of reduced species at large depth that is not considered in the model due to the transient and locally restricted nature of these events. Transient re-oxidation of reduced substances is also indicated by elevated concentration of Fe(III)HCl (i.e,, poorly crystalline Fe oxide minerals) in subsurface sediments (Supplementary Figure S3) and from the enrichment of microaerophilic Fe(II)-oxidizing bacteria from the sediments (Supplementary Figures S11, S12). Poorly crystalline Fe(III) minerals undergo rapid conversion to pyrite (Canfield et al., 1992) and are, thus, not stable under the highly sulfidic conditions prevailing at this depth.

Reaction rates were plotted in Supplementary Figures S4, S5 and listed in the Supplementary Table S2. Depth-integrated POC degradation, SRR, SOR, AOM and rates for methanogenesis were in the same order of magnitude as those previously reported for Boknis Eck sediments, albeit some differences were noted (Treude et al., 2005; Preisler et al., 2007; Bertics et al., 2013; Dale et al., 2013; Maltby et al., 2018). Considerably higher rates modeled for October 2018 relative to March 2019 sediments were in line with higher bottom water temperatures (Supplementary Figure S13). The picture that emerges from the rate vs. depth plot indicates that the highest rates are encountered in the surface layer where POC is degraded and sulfide is oxidized by a range of processes (see Supplementary Results and Discussion). This result is consistent with a large range of observations showing that fresh phytoplankton biomass deposited at the sediment surface is much more reactive than sedimentary OM buried below. The model also predicts that SR and methanogenesis occurred in the surface layer, which is again due to the high reactivity and large depositional POC flux at the surface that allows for an overlap of different processes that are otherwise spatially separated. SR is the dominant process in the underlying sediment section followed by methanogenesis in the sulfate-depleted section at the base of the model column. AOM rates exceeded rates of methanogenesis since large amounts of methane appear to be delivered across the lower boundary of the model *via* upward diffusion. Hence, most of the AOM is driven by upwards diffusing methane rather than methanogenesis within the model column (reaching down to 40 cm) and is likely fueled by the large stock of sedimentary POM that has accumulated during higher eutrophication periods in the past. Noticeable is also that the peaks of modeled SOR, SRR, AOM and methanogenesis are shallower for sediments from October 2018 than for those from March 2019 (Figures 3, 4; see also Supplementary Results and Discussion). Moreover, higher sediment temperatures in October 2018 compared to March 2019 induced an increase in SR and methanogenesis such that more reduced metabolites (hydrogen sulfide and methane) were formed and transported towards the sediment surface.

#### Vertical zonation of active prokaryotes (RNA-profiling) in sediments

As expected, cell numbers in sediments of both studied seasons and years decreased with depth (Figure 2A). For October 2018, less bacterial cells were active in the deeper sediments than in the surficial sediment regions, for March 2019 this was not the case (Figure 2B). According to CARD-FISH, in October 2018 active bacterial cells decreased considerably from 74% (2–3 cm) to 58% (20–22 cm), while active archaeal cells increased from 3 to 11% with depth (Figure 2B). The same approach demonstrated only minor changes between the relative abundance of Bacteria (71 to 70% for the same depth horizons) for March 2019 but an increase of the archaeal proportion from 3 to 9% (Figure 2B). The relative abundance between Bacteria and Archaea, as documented by qPCR, showed the same trend: Archaea increased with depth from 1 to 11% for October 2018 and to 4% in March 2019.

The microbial community compositions and the metabolic potentials identified for the different taxa in the Boknis Eck sediments largely followed the vertical stratification guided by the redox cascade commonly observed in marine sediments (*cf*. Middelburg, 2018). According to changes in community compositions based on 16S tags from transcripts, three depth zones were distinguished (Supplementary Figures S6A–D, S7A,B, details in Supplementary results and discussion): Zone 1 marked the highly reactive surface layer hosting a wide range of aerobic and anaerobic microorganisms, the underlying anoxic zone 2 was dominated by fermenting and sulfate reducing microbes, while the deepest zone 3 hosted microbial consortia conducting AOM. Zone 1 (0–2 cm) was typically hallmarked by autotrophic sulfide-oxidizing Beggiatoales (primarily *Candidatus* Isobeggiatoa, *Candidatus* Parabeggiatoa) (Bacteria) (missing in MIC1 10/2018) and aerobic ammonia-oxidizing Nitrosopumilales (Archaea) as prominent clades (Supplementary Figures S6A–D, S7A,B). In the October 2018 sediments Nitrosopumilales and Beggiatoales composed up to 32 and 13% of the archaeal and bacterial 16S tags of transcripts, respectively. In contrast, in the 2019 sediments Beggiatoales were a very prominent bacterial clade (46%) and *Nitrosopumilus* reached maxima of 18%. This layer, as well as zone 2 was accompanied by a mix of organotrophs, including mostly fermenters producing H_2_, CO_2_, and acetate, sulfate-reducing Bacteria (SRB) (primarily SRB-SEEP1 of the *Desulfobacteria*) as well as a phylogenetically diverse mix of potential sulfide-oxidizing (autotrophic) Bacteria (Supplementary Figures S6A–D). These Bacteria probably rely on oxygen that is episodically transported to large sediment depth - as indicated by the disturbed chloride profile in MIC2 (Supplementary Figure S9) and the presence of poorly crystalline Fe (oxyhydr)oxides in subsurface sediments (Supplementary Figure S3). In zone 3 the potential SRB shifted from mostly uncultured SRB-SEEP1 (prevailing in zone 1 and 2) to *Desulfatiglans* (up to 20%; Supplementary Figures S6A–D).

Based on 16S tags from transcripts, considerably less archaeal than bacterial taxa were involved in the vertical shifts. In all depth horizons Archaea of the Bathyarchaeia, Methanofastdiosa, Woesearchaeota, Lokiarchaeota, and marine benthic Group D/DHVEG-1 were identified in different proportions (Supplementary Figures S7A,B). These members are potentially associated with versatile metabolisms including thiosulfate oxidation, acetogenesis, fermentation, methylotrophic methanogenesis, hydrogen-dependent carbon dioxide fixation as well as dissimilatory nitrite and SR (Supplementary results and discussion). *Candidatus* “Methanofastidiosa” seemed to be displaced by potential ammonia-oxidizing *Nitrosopumilus* in zone 1 and by ANME-1 and ANME-2 (ANaerobic MEthane oxidizers) in zone 3.

The above-described vertical zones 2 and 3 were found in all sediment cores but varied in depth depending on the time of the year and likely environmental conditions. The zone 3, where modeled AOM rates peaked, was generally shallower in the October 2018 than the March 2019 samples (Supplementary Figures S6A–D, S7A,B).

### Seasonal dynamics of biologically controlled sulfide and methane turnover rates and RNA-profiling for October 2018 and March 2019

When comparing the modeled rates with the taxa distributions (Figures 3, 4) and cell numbers (Figure 2) two things stand out: (i) the modeled rates were considerably higher for October 2018 than for March 2019 sediments, matching markedly higher cell numbers for 2018 and (ii) the modeled maxima for SOR, SRR, rates of AOM, and methanogenesis were shallower for October 2018 than for March 2019, consistent with vertical shifts of taxa defining vertical zones.

#### Higher sulfide and methane production and oxidation rates in October 2018 than in March 2019

Modeled SRR maxima in the first 2 cm were about 10-fold higher for October 2018 than for March 2019 (Figures 3A,B). In these depth ranges 16S tags of transcripts related to SRB were up to 3-fold higher and total cell numbers up to 20% higher for the October 2018 core. The estimated per cell SRR for October 2018 were 6-fold higher in the surface and 2-fold higher in zone 3 relative to the March 2019 sediment (Supplementary Figure S14) supporting a higher sulfide flux from sediments in fall 2018. Relative to previously measured SRR (Treude et al., 2005; Bertics et al., 2013), the more recent modeled SRR (Figures 3A,B) appear to be up to 30% higher.

Modeled maxima of SOR were about 3-fold higher in October 2018 (Figures 4A,B), matching the higher suggested sulfide fluxes. The estimated per cell SOR were 20-fold higher in October 2018 than in March 2019 in the 0–1 cm sediment horizons, but below 1 cm were quite comparable for the two seasons (Supplementary Figure S14). Hence, with the higher sulfide flux in October 2018, it is not surprising that below 1 cm, sulfide was accumulating in October 2018 faster and in shallower sediment regions than in March 2019 (Figures 1A,C). In March 2019, *Beggiatoaceae* dominated the top 2 cm sediment layers, while in October they only made up 4% of the 16S tags from transcripts (Figures 3A,B; Supplementary Figure S6). Work at Boknis Eck dating back 16 years, already suggested that the abundant *Beggiatoaceae* only accounted for a comparatively small fraction of the sulfide removal in the sediments (Preisler et al., 2007). However, *Beggiatoaceae* also actively avoids too high sulfidic conditions (Dunker et al., 2011), albeit requiring sulfide as an electron donor. This compares with their presence linked to lower sulfide fluxes in March 2019, while higher sulfide fluxes in October 2018 were reflected rather by a phylogenetically more diverse potential sulfide oxidizing community as indicated by the presence of, e.g., uncultured *Thiotrichaceae*, *Ectothiorhodospiraceae*, *Sulfurospirillaceae*, *Sulfurovaceae* and *Sulfurimonadaceae* (Figure 4). In summary, this indicates that in October 2018 the sulfide oxidizing community was phylogenetically more diverse and exhibited higher SO turnover rates than in March 2019.

Methanogenesis rates were roughly 11-fold (Figures 4C,D) higher in October 2018 than in March 2019 sediments. The methanogenesis peaks coincided with an enrichment of Woesarchaeota and *Nitrosopumilus* 16S tags of transcripts (Figures 4C,D). Neither taxon is known to catalyze methanogenesis. Although Woesearchaeota co-occurrence network analyses have implied a syntrophic relationship of Woesearchaeota with methanogens (Liu et al., 2018). Interestingly, modeled methanogenesis peaks also co-occurred with modeled SRR peaks (discussion on competitive and non-competitive substrates in Supplementary Material).

Modeled AOM rates peaking in and below 20 cm were two-fold higher in October 2018 (Figures 3C,D). At this depth range total cell numbers for the two seasons were relatively similar, but ANME-1b 16S tags of transcripts had a nearly two-fold higher relative abundance in the October 2018 sediments at 20–22 cm depth (Figures 3C,D). Estimated per cell AOM rates remained comparable for both tested years and depths (Supplementary Figure S14) but given that more methane consuming Archaea were present in October 2018, the total methane consumption can be considered higher. For more discussions on estimated per cell rates see the Supplementary Material. The higher modeled rates for the October 2018 sediments were likely related to elevated sediment temperatures (Supplementary Figure S15) and the fresh supply of POC following the phytoplankton bloom.

#### A shallower biological sulfide and methane filter in October 2018 than in March 2019

The maxima of the modeled SOR, SRR, AOM, and methanogenesis rates were shallower in October 2018 than in March 2019 (Figures 3, 4; Supplementary Figures S4, S5). Correspondingly, taxa known to catalyze the respective chemical reactions, i.e., SRB and ANME were also in shallower depth horizons (Supplementary Figures S6, S7). Temperature plays a major role for stimulating metabolic processes and methanogenesis derived methane emission is markedly increased with temperature (e.g., Yvon-Durocher et al., 2014; Jansen et al., 2022). Bottom water temperatures for October 2018 were higher (*t* = 13°C) than in March 2019 (*t* = 5°C; Supplementary Figure S1) and likewise were modeled sediment temperatures (Supplementary Figure S15), stimulating methane production. The higher methane concentration in the sediments fuel AOM, which in turn stimulates SRR, producing more sulfide, which can be utilized by sulfide oxidizing Bacteria. For Boknis Eck, this scenario results in potential sulfate reducing and sulfide oxidizing Bacteria and methane oxidizing Archaea as well as their respective modeled rates (Figures 3, 4) alongside with more produced alkalinity being located in shallower regions in October 2018 than in March 2019 (Figures 1D,F). Similarly, a recent study has suggested that increased temperature amplified negative effects at the base of coastal biogeochemical cycling (Seidel et al., 2022). In that study elevated temperatures, reduced oxygen concentrations alongside with decreased electron acceptor availability and higher SRR, moved anaerobic reactions and more diversified microbes closer to the sediment-water-interface (Seidel et al., 2022).

Despite these obvious dynamics between the two tested seasons, we did not observe significant differences (value of *p* <0.01) between the overall bacterial communities from October 2018 and March 2019 based on 16S tags from transcripts. Yet, analyses only consider the community across the core as a whole, but not the vertical changes within the cores. However, a significant difference was observed between the archaeal communities based on RNA-profiling for the two seasons (value of p <0.01) suggesting that the Archaea reacted to environmental changes by altering their community, while the Bacteria appeared to primarily act by shifting vertically.

### Effects of biologically controlled emissions based on RNA- and DNA-profiling

There were several significant differences (value of *p* <0.001) between the RNA- and DNA-based 16S tag communities. These likely also reflected the different degrees of stability of microbial DNA (hours to days and up to years and even millennia under favorable conditions) and RNA (minutes) once released from the cells into the environment (Torti et al., 2015; Ottesen, 2016). Additionally, DNA-based profiling targets living and dead cells as well as extrachromosomal DNA, whereas RNA detects only metabolically active and dormant cells (see Supplementary results and discussion). However, when comparing data derived from DNA-profiling with that of RNA-profiling, some general uncertainties need to be considered, which include the ratio between rRNA and DNA encoding rRNA genes in the environmental sample, expression levels and copy number of 16S rRNA genes, and different genome sizes.

#### 16S tag enrichments in RNA

The most abundant Bacteria and Archaea marking significant differences between the DNA-based and RNA-based community profiling are highlighted in Figure 5. The 16S tags enriched from transcripts relative to those from DNA likely reflect the current advantageous environmental conditions for members of these phylotypes. For Bacteria, these primarily included members of the organotrophic *Pirellulaceae*, methane oxidizing *Methylomonadaceae* and potentially autotrophic sulfide oxidizers like *Thiotrichaceae*, *Sulfurimonadaceae*, *Ectothiorhodospiraceae* and to a lesser extent also *Chromatiaceae* (Figure 5A). This suggests that environmental conditions have changed over time to support phylogenetically more diverse sulfide oxidizing microbes apparently (as shown above) turning over more sulfide.

The Archaea that were significantly enriched in RNA over DNA included mostly *Methanofastidiosales*, putatively operating as obligate H_2_-dependent methylotrophic methanogens (Vanwonterghem et al., 2016), Odinarchaeia, anaerobic heterotrophs (MacLeod et al., 2019), Bathyarchaeia (Zhou et al., 2018) and anaerobic methane oxidizing *Methanosarcinales* (ANME; Kendall and Boone, 2006; Figure 5B; Supplementary Figure S7). The versatile metabolisms of the anaerobic Bathyarchaeia include acetogenesis, methane metabolism, as well as dissimilatory nitrite and SR (Zhou et al., 2018). They can utilize detrital proteins, polymeric carbohydrates, fatty acids, aromatic compounds, methane (or short chain alkane) and methylated compounds. The enrichment of ANME in the RNA-profiling supports the above-mentioned strengthening of the biological methane turnover in these sediments.

#### 16S tag enrichments in DNA

In contrast, the 16S tags strongly enriched in DNA but depleted in the RNA were likely relicts from a previous phase where environmental conditions were more beneficial for respective taxa. These DNA based 16S tags resembled mainly organotrophs, i.e., *Anaerolineaceae* (Chloroflexi), *Desulfobulbaceae*, *Calditrichaceae*, *Woesiaceae*, and *Flavobacteriaceae* as well as one group of sulfur oxidizers, i.e., *Sulfurovaceae* (Figure 5A). According to RNA based 16S tags, members of different *Desulfobacteriaceae* primarily dominated the SRB in the sediment cores. DNA based 16S tags from zone 2, identified additionally *Desulfuromonadia* (an elemental sulfur reducer), *Desulfocapsa* (disproportionates elemental sulfur and thiosulfate) and uncultured *Desulfobulbaceae* for October 2018 and *Desulfobulbaceae* in March 2019 on a level of significance (data not shown). This discrepancy between 16S tags derived from RNA and DNA could be explained by temporal modifications in environmental conditions, not just reflected by seasonality, favoring different organic degradation metabolisms of distinct SRB. For example, *Desulfobulbaceae* - recognized in both seasons and by RNA and DNA - can directly oxidize substrates released from extracellular hydrolysis like sugars and amino acids (Kuever, 2014) and have to compete with fermenters for these substrates. *Flavobacteriaceae* utilize macromolecules such as polysaccharides and proteins (Mcbride, 2014). Most members of *Anaerolineaceae* fermentatively utilize sugars and proteinaceous compounds. Although external electron acceptors are not observed for their growth they can use hydrogenotrophs as a hydrogen(electron)-scavenging system (Yamada and Sekiguchi, 2018). *Calditrichaceae* ferment peptides or implement nitrate reduction with acetate or molecular hydrogen as electron donors (Kublanov et al., 2017). One interpretation of this DNA–RNA data comparison can be that over longer time scales the sedimentary community has changed from phylogenetically diverse organotrophic microbes to phylogenetically diverse autotrophic sulfide oxidizers.

The reduced eutrophication at Boknis Eck during the last decades has caused lower primary production (Lennartz et al., 2014) and may result in less POM reaching the sediments. Also, POM composition appears to have changed in the years 2015 to 2019 (Supplementary Figure S2). In parallel, in the last decades bottom water temperatures have risen and oxygen concentrations declined (Lennartz et al., 2014), likely stimulating temperature-dependent methanogenesis (e.g., Yvon-Durocher et al., 2014; Jansen et al., 2022). Elevated methane production promotes higher SRR, which in fact appear to have increased since 2000 (Treude et al., 2005). Stimulated sulfide production, causes the system to exhibit a high sulfide flux and an increase in SOR. The environmental changes of the Boknis Eck system over longer than seasonal time scales could explain the significant differences (value of *p* is 0.001) between the microbial communities based on DNA-and RNA-profiling. More work including *in situ* and laboratory experiments alongside with long-term monitoring of the sedimentary community will be necessary to better understand how the observed increase in benthic methane and sulfide turnover may affect the release of these reduced substances into the overlying bottom water.

## Conclusion

Benthic production and oxidation of sulfide and methane were accelerated in October 2018 relative to March 2019. These seasonal trends can be largely explained by elevated bottom water and sediment temperatures during October that stimulated POM degradation *via* SR and methanogenesis. The increase in methane production induced a corresponding rise in AOM, while sulfide oxidation was accelerated since more sulfide was formed *via* POM degradation and AOM.

The long-term trends in local pelagic environment conditions are in line with the differences observed between benthic DNA-and RNA-profiling of 16S tags. Although such DNA/RNA comparisons entail potential limitations, as discussed above, the differences in benthic microbial community compositions can be interpreted as a marked response induced by temporal, non-seasonal environmental modifications. The observed decline in pelagic productivity is responsible for less reactive POM delivered to the sediment by local phytoplankton such that a relative decline in 16S tags of transcripts related to organotrophs could reflect diminishing activity of microbes degrading fresh phytoplankton.

Coeval bottom water warming enhanced microbial processing of the large stock of sedimentary POM that accumulated during the previous eutrophication phase. This led to an increase in sulfide production and a corresponding rise in sulfide oxidation that is observed as a relative increase in 16S tags of transcripts related to sulfide oxidizing microorganisms. Sulfide producing processes may have been further accelerated by the decline in dissolved oxygen in ambient bottom waters favoring anaerobic POM degradation processes. The overall shift from POM degrading to sulfide oxidizing Bacteria could reflect, a decline in fresh POM availability, a temperature-driven acceleration in sedimentary POM turnover and an increase in sedimentary anoxia induced by bottom water deoxygenation.

The upward shift of the sulfide oxidation layer towards the sediment surface suggested by historic pore water profiles probably reflects the general increase in sulfide production discussed above that induced an increase in the upward diffusive flux of sulfide and, thereby, a shoaling of the sulfide oxidation layer. If these trends would continue into the future, they may induce a rise in toxic sulfide release from sediments with potentially harmful consequences for pelagic ecosystems.

## Data availability statement

The datasets presented in this study can be found in online repositories. The names of the repository/repositories and accession number(s) can be found in the article/Supplementary material.

## Author contributions

MP designed microbiological study, interpreted data, and developed and wrote manuscript. KW modeled rates, interpreted data, and developed manuscript. NA-B and KL-M performed molecular biology work and bioinformatics analyses. SB performed qPCR and monitored cell counts. ID performed CARD-FISH. HB performed geochemical analyses of water column parameters. DI carried out the sequencing. VN, CB, and AK performed MPN counts of Fe metabolizing microbes. HH and AE conducted flow cytometry, total dissolved amino acids, and DOC in the water column. FS led sampling campaign, geochemical pore water and solid phase analyses and their interpretation. All authors contributed to interpretations and manuscript writing.

## Funding

The methane measurements were part of the BONUS INTEGRAL project which received funding from BONUS (Art 185), funded jointly by the EU, the German Federal Ministry of Education and Research, Swedish Research Council Formas, Academy of Finland, the Polish National Centre for Research and Development, and the Estonian Research Council. Water column measurements of nano-and pico-plankton as well as TDAA and DOC were part of the project CREATE (grant no. 03F0910A) funded by the German Federal Ministry of Education and Research. Sediment geochemistry analyses were funded by the German Research Foundation (DFG) through Emmy Noether Project ICONOX to FS.

## Conflict of interest

The authors declare that the research was conducted in the absence of any commercial or financial relationships that could be construed as a potential conflict of interest.

## Publisher’s note

All claims expressed in this article are solely those of the authors and do not necessarily represent those of their affiliated organizations, or those of the publisher, the editors and the reviewers. Any product that may be evaluated in this article, or claim that may be made by its manufacturer, is not guaranteed or endorsed by the publisher.

## Footnotes

^1^ www.bokniseck.de
