# Supplementary material for: Environmental changes affect the microbial release of hydrogen sulfide and methane from sediments at Boknis Eck (SW Baltic Sea)
Source: Front Microbiol. 2022 Dec 21;13:1096062. doi: 10.3389/fmicb.2022.1096062 (PMC9822571; doi:10.3389/fmicb.2022.1096062)
Supplement: Supplementary file 1 [file Data_Sheet_1.docx]

**Supplementary Information for**

**Environmental changes affect the microbial release of hydrogen sulfide and methane in sediments at Boknis Eck (SW Baltic Sea)**

Mirjam Perner^1^*, Klaus Wallmann^1^, Nicole Adam-Beyer^1^, Helmke Hepach^1^, Katja Laufer-Meiser^1^, Stefanie Böhnke^1^, Isabel Diercks^1^, Hermann W. Bange^1^, Daniela Indenbirken^2^, Verena Nikeleit^3^, Casey Bryce^3,4^, Andreas Kappler^3^, Anja Engel^1^, Florian Scholz^1^

^1^GEOMAR Helmholtz Centre for Ocean Research, Department of Marine Biogeochemistry, Kiel, Germany

^2^Leibniz Institute of Virology, Martinistraße 52, 20251 Hamburg, Germany

^3^Geomicrobiology, Department of Geosciences, University of Tübingen, Tübingen, Germany.

^4^School of Earth Sciences, University of Bristol, Bristol, UK.

**This file contains:**

Supplementary Material and Methods S1-S4

Supplementary Results and Discussions

Supplementary tables S1 - S5

Supplementary figure S1 - figure S17

*Corresponding Author: Mirjam Perner, GEOMAR Helmholtz Centre for Ocean Research Kiel, Geomicrobiology, Wischhofstraße 1-3, 24148 Kiel, Germany, Tel: +49-431-600-2837, Fax: +49-431-600-2941, E-mail: mperner@geomar.de

**Supplementary Material and Methods**

**Supplementary Material and Methods S1: Sample collection and sample analyses**

Cores for microbiological analyses were retrieved from two distinct deployments each in 2018 and 2019 (D1 and D2). Adjacent sediment cores were collected for geochemical analyses (pore water chemistry and solid phase analyses), with the exception of the 2019 campaign, where only one adjacent core (from D1) could be recovered (see Supplementary table S1). The cores varied in length from 22 to 26 cm and were subsampled at a resolution of 1 cm in the first 6 cm, followed by 2 cm-wise sampling below the 6 cm horizon. DNA was extracted from four cores (MIC1 and MIC3 in 2018 and 2019) and bacterial and archaeal 16S tags compiled. RNA was also extracted and bacterial 16S tags were generated from these four cores as well but archaeal 16S tags only from one core of each year and season (MIC1 in 2018 and 2019). Cell numbers were determined for the same MIC1 cores in 2018 and 2019. QPCR, i.e. quantitative analyses of Bacteria relative to Archaea, were performed for the 2-3 cm and for the 20-22 cm depth horizons for MIC1 of October 2018 and for MIC1 in March 2019, representative of the surface/intermediate and the AOM (anaerobic oxidation of methane) zone.

**Supplementary Material and Methods S2: Geochemistry of the water column, sediments, and pore waters**

Water samples were taken from six water depths (1, 5, 10, 15, 20 and 25 m) with a CTD/Rosette equipped with 5L Niskin bottles. Nutrient and oxygen concentrations were measured according to the standard methods described in (Grasshoff et al., 1999). Methane concentrations were measured with a static equilibration method and a gas chromatograph equipped with a flame ionization detector. The details of the method can be found in Ma et al. (2020).

Sediment cores were retrieved with a minicorer (MIC). The bottom water overlying the sediment in the MIC liners was siphoned with a plastic tube and filtered for subsequent analyses. To prevent oxidation of reduced substances, sediment cores were subsampled in a glove bag under argon atmosphere at a resolution increasing from 1 cm at the surface to 4 cm at greater depth. A defined volume of sediment was sampled into pre-weighed plastic cups for the determination of water content and porosity. Pore water was separated from the solid phase by centrifuging sediment samples for 15 minutes at 4000 rpm. The supernatant pore water samples were filtered (0.2 µm cellulose-acetate filters) under argon atmosphere in a second glove bag. Subsamples for metal analyses were acidified with concentrated nitric acid (sub-boiled distilled).

Sediment samples in centrifuge tubes were kept for shore-based analyses of the solid phase. Hydrogen sulfide concentrations were determined photometrically using the methylene blue method (Grasshoff et al., 1999). Total alkalinity was analyzed by titration of 0.25 - 1 ml pore water with 0.02 M HCl according to Ivanenkov and Lyakhin (1978). During titration, the sample was degassed by continuously bubbling nitrogen to remove any generated CO_2_ and H_2_S. The results were calibrated relative to IAPSO seawater solution. Concentrations of manganese (Mn) and iron (Fe) were determined by inductively coupled plasma optical emission spectroscopy (ICP-OES) or mass spectrometry (ICP-MS; Mn < 5 µM, Fe < 1 µM). Sulfate concentrations were measured by ion chromatography.

The distribution of Fe oxide and sulfide minerals in the sediment (together referred to as reactive Fe) were determined by sequential extraction of operationally defined sedimentary Fe pools. In the first step, wet sediments in centrifuge tubes were treated with 0.5 M HCl in an overhead shaker for one hour followed by centrifugation. Ferrous Fe (Fe(II)) in the extraction solution was analyzed photometrically using Ferrozine (Stookey, 1970) and total Fe was analyzed by inductively coupled optical emission spectrometry (ICP-OES). These fractions comprise Fe bound to Fe(II) monosulfide (Fe(II)_HCl_) and poorly crystalline Fe(III) (oxyhydr)oxide (Fe(III)_HCl_ = Fe_HCl_ – Fe(II)_HCl_; e.g., ferrihydrite) (Kostka and Luther, 1994). Crystalline Fe(III) (oxyhydr)oxide (e.g., goethite) was extracted using sodium dithionite buffered to pH 4.8 using acetic acid and sodium citrate (Fe_dith_) (Kostka and Luther, 1994). Concentrations of Fe bound to pyrite (FeS_2_; Fe_py_) were determined by the chromium reduction method (Canfield et al., 1986). The water content of wet sediments was determined separately for the calculation of concentrations per dry weight. The extent to which Fe oxides have been converted to Fe sulfide minerals is defined as (Fe(II)_HCl_ + Fe_py_)/(Fe_HCl_+Fe_dith_+Fe_py_).

**Supplementary Material and Methods S3: Modeled rates**

The 1-D model considers the inert tracer chloride and the reactive species sulfate, sulfide and methane. Since molecular diffusion constants and reaction rates depend on temperature, we also simulate the time-dependent temperature distribution in sediments. The model considers molecular diffusion, sediment burial and compaction as transport processes for dissolved tracers.

Most of the biogeochemical processes considered in the model are driven by the microbial degradation of particulate organic carbon (POC). The rate of POC degradation (R_POC_) steeply declines with sediment depth as usually observed in marine surface sediments (Middelburg, 1989). The R_POC_ depth profile is prescribed using an empirical rate law (Stolpovsky et al., 2015) and the annual mean rain rate of POC observed at Boknis Eck (Dale et al., 2013). R_POC_ also increases with temperature since organic matter degradation and other microbial processes proceed more rapidly under elevated temperatures in our study area (Treude et al., 2005;Dale et al., 2013). At the sediment surface, POC is degraded using oxygen, nitrate, nitrite, MnO_2_ and FeOOH as terminal electron acceptors. These degradation pathways are lumped into one rate (R_O_) since our data set does not allow for the resolution of these overlapping processes. The prescribed thickness of the oxic to suboxic surface layer varies between 1.5–2.0 cm during the warm summer period and 4 cm in April 2018 when cold water masses entered our study area (supplementary figure S13). Below the surface layer, POC is first degraded via dissimilatory sulfate reduction (R_SR_) and subsequently via methanogenesis after sulfate is depleted (R_M_).

In addition to primary processes that are directly driven by POC degradation (R_O_, R_SR_, R_M_), the model also considered secondary redox reactions where reduced species formed via anaerobic POC degradation (R_SR_, R_M_) are re-oxidized within the sediment column. Sulfide formed via R_SR_ and R_M_ diffuses toward the surface where it is oxidized within the oxic - suboxic surface layer. All possible sulfide oxidation reactions are again lumped into one reaction term (R_SO_). Methane formed via R_M_ in sulfate-depleted sediment sections and methane ascending across the lower boundary of the model are consumed by AOM at sediment depths where upwards diffusing methane meets downward diffusing sulfate. In the model, sulfate is used as the sole electron acceptor for AOM (R_AOM_).

The model is run only over a short period of time from Jan. 2018 to March 2019. Hence, the model results depend critically on the initial conditions chosen for Jan 2018. We employed tracer concentrations that have been previously measured in Boknis Eck sediments during the winter season to defined the initial depth profiles for all dissolved species consider in the model (Abegg and Anderson, 1997;Treude et al., 2005;Dale et al., 2013).

Time-dependent bottom water temperatures and concentrations are prescribed at the upper boundary of the model located at x = 0. The monitoring data obtained at Boknis Eck from Jan. 2018 till March 2019 show strong a temporal variability in temperature and salinity at 25 m water depth (supplementary figure S13). Salinity data are used to calculate time-dependent chloride and sulfate concentrations as boundary condition using the ratios between salinity and major anion concentrations in seawater (Millero, 1996). Methane and sulfide concentrations are set to zero for x = 0.

The lower boundary of the model domain is located at 100 cm sediment depth. Zero gradient conditions are used for the dissolved tracers while a constant gradient, corresponding to the local geothermal gradient, is applied for temperature. In the following, we only show model results for the upper 40 cm of the model column.

***Model set-up.*** A non-steady state model was set up to evaluate porewater data from Boknis Eck. The model considers the inert tracer chloride and the reactive species sulfate, sulfide and methane. Since molecular diffusion constants and reaction rates depend on temperature, we also simulate the temperature distribution in sediments.

The following heat equation was set up and solved for temperature simulations:

$\frac{d H_{B} T}{d t}=\frac{d}{d x}\left( \lambda_{B} \frac{d T}{d x} - \left( v_{W} H_{W} + v_{S} H_{S} \right) T \right)$ (1)

where H_B_ is heat capacity of bulk sediment, T is temperature, λ_B_ is thermal conductivity of bulk sediment, v_W_ is burial velocity of porewater, v_S_ is burial velocity of solids, H_W_ is heat capacity of porewater, H_S_ is heat capacity of solids, t is time and x is sediment depth.

For dissolved tracers, the partial differential equation is formulated as:

$\frac{d P C}{d t}=\frac{d}{d x}\left( P D_{S} \frac{d C}{d x} - v_{W} P C \right)+a P \left( C_{BW} - C \right) + P R$ (2)

where C is the concentration of the considered tracer in porewater, P is porosity, D_S_ is the diffusion coefficient in sediments, a is the bio-irrigation coefficient, C_BW_ is the tracer concentration in ambient bottom water and R is the reaction rate. The diffusion coefficients depend on temperature and porosity. They are calculated for each species using equations given in (Boudreau, 1997). To consider charge coupling, the harmonic mean of the diffusion coefficients of Na^+^ and Cl^-^ is applied in the model to simulate the diffusive transport of Cl^-^ (Cussler, 1984).

Reactions, kinetic rate laws and other functions considered in the model are listed in supplementary table S3. The kinetic rate law for the degradation of particulate organic carbon (R_POC_) is taken from (Stolpovsky et al., 2015). This empirical power law prescribes the steep down-core decrease in R_POC_ usually observed in marine surface sediments. The rate equation also includes a temperature term since organic matter degradation and other microbial processes proceed more rapidly under elevated temperatures in our study area (Treude et al., 2005;Dale et al., 2013). In a thin surface layer, POC is degraded using oxygen, nitrate, nitrite, MnO_2_ and FeOOH as terminal electron acceptors. These degradation pathways are lumped into one rate (R_O_) since our data set does not allow for the resolution of these overlapping processes. A simple Boltzmann function (f_O_) is applied to define the thickness of this oxic to suboxic surface layer where it is assumed that the thickness decreases with increasing bottom water temperature (T_BW_). Below the surface layer, POC is first degraded via dissimilatory sulfate reduction (R_SR_) and subsequently via methanogenesis (R_M_). Sulfide formed at depth diffuses toward the surface where it is oxidized using a range of electron acceptors. All possible sulfide oxidation reactions are again lumped into one term (R_SO_). Methane ascending from depth is consumed by anaerobic oxidation of methane close to the base of the investigated sediment cores (R_AOM_).

All parameter values applied in the model are listed in supplementary table S4. Rate terms that are applied in Eq. 2 (R) for each of the considered species are shown in supplementary table S5. They reflect the stoichiometry of the considered microbial processes.

***Initial and boundary conditions.*** Initial conditions are applied to define tracer distributions at t = 0. A linear profile was applied for temperature:

$T_{i}=T_{BW}(0)+ T_{GR} x$ (3)

where T_BW_(0) is the bottom water temperature at t = 0 and T_GR_ is the local geothermal gradient (30 °C km^-1^).

For dissolved chloride, we employ a simple exponential function:

$C_{i}=\left( C_{BW}(0) - C_{F} \right) e^{- 0.2 x} + C_{F}$ (4)

where C_i_ is the concentration at t = 0, C_BW_(0) is the bottom water concentration at t = 0, and C_F_ is the concentration at large sediment depth at t = 0 (353 mM).

For sulfate, we applied a function that mimics the steep exponential decline that is representative for our site:

$C_{i}=C_{BW}(0)e^{- 1/3 x}$ (5)

A Boltzmann function was employed for methane and sulfide:

$C_{i}=C_{F} - \frac{C_{F}}{1+e^{\frac{x -x_{M}}{2}}}$ (6)

The asymptotic concentration at large depth (C_F_) at t = 0 was set to 4 mM for sulfide and 7.2 mM for methane while x_M_ = 10 cm was applied for sulfide and x_M_ = 30 cm for methane. The C_F_ value for methane corresponds to the solubility of methane gas for the initial temperature profile and is consistent with methane concentrations measured in sediments retrieved with a pressurized core barrel at a nearby site (Abegg and Anderson, 1997).

Time-dependent bottom water temperatures and concentrations are prescribed at the upper boundary of the model located at x = 0. The monitoring data obtained at Boknis Eck from Jan. 2018 till March 2019 show strong a temporal variability in temperature and salinity at 25 m water depth (supplementary figure S13). Salinity data are used to calculate time-dependent chloride and sulfate concentrations as boundary condition using the ratios between salinity and major anion concentrations in seawater (Millero, 1996). Methane and sulfide concentrations are set to zero for x = 0.

The lower boundary of the model domain is located at 100 cm sediment depth. Zero gradient conditions are used for the dissolved tracers while a constant gradient, corresponding to the geothermal gradient is applied for temperature. In the following, we only show model results for the upper 40 cm of the model column.

The model was solved using the PDE solver of Mathematica, version 13. The solver uses the method-of-lines approach on an uneven grid with a high resolution at the surface (0.001 cm), a coarser resolution at depth (0.2 cm) and a total of 1000 depth layers. The model period extends from Jan 1^st^, 2018 till March 31^st^, 2019.

**Supplementary Material and Methods S4: Microbiology**

***DNA and RNA extraction, cDNA synthesis, amplicon library preparations and Illumina sequencing.*** DNA was extracted from around 300 mg sediment using the NucleoSpin DNA Soil Mini kit (53 and 139 ng µl^-1^ with a 260/280 nm ratio ranging from 1.7 to 1.8) (Macherey-Nagel, Düren, Germany) according to the specifications of the manufacturer. For RNA isolation approximately 0.25 mg sediment and the NucleoBond RNA Soil Mini kit (Macherey-Nagel) following the manufacturer’s instructions were used. Subsequently DNaseI digestion was done by using the RapidOut DNA Removal kit (Thermo Fisher Scientific GmbH, Waltham, Massachusetts, USA), according to the provided protocol (7.1 and 186 ng µl^-1^ with a 260/280 nm ratio ranging from 1.6 to 2.0). Isolated RNA was transcribed into cDNA with Invitrogen’s SuperScript^®^ VILO^TM^ cDNA Synthesis Kit (Life Technologies^TM^, Darmstadt, Germany), according to the manufacturer's protocol. Purified cDNA (Zymo Clean & Concentrator-5, Irvine, CA, USA) was used as a template to create a 16S metagenomic sequencing library compatible with the Illumina MiSeq system as specified in the manufacturer's guideline (Illumina®, San Diego, CA, USA). The procedures have been described before : 16S ribosomal RNA and DNA gene amplicons were generated for the (i) bacterial hypervariable V3 and V4 regions using the primer pair S-D-Bac-0341-b-S-17 and S-D-Bac-0785-a-A-21 (Klindworth et al., 2013) and for the (ii) archaeal hypervariable V4 and V5 regions using the two primer pairs (i) Arch_519F and Arch 915R, (Ding et al., 2017) and (ii) Arch 524F and Arch 958R (Cerqueira et al., 2017). Two parallel PCR reactions were performed for each of the three primer sets (in total 3x2 PCRs per sample) using the Kapa Hifi HotStart Ready Mix (Kapa Biosystems, Boston, MA, USA) as specified in the manufacturer’s protocol. PCR conditions were set as follows: 3 min initial denaturation followed by 25 cycles of denaturation at 95°C for 30 s, annealing at 55°C and 63°C for amplification of the bacterial and the archaeal 16S rRNA gene, respectively, and extension at 72°C for 30 s. After pooling the 1x2 parallel samples of the bacterial 16s rRNA gene amplification and the 2x2 parallel samples of the archaeal 16S rRNA amplification purification was done using the Agencourt AMPure XP beads (Beckman Coulter, Brea, CA, USA) according to manufacturer’s protocol. In a subsequent amplification step multiplexing indices and Illumina^®^ sequencing adapters were added by using the Nextera^®^ XT Index Kit (Illumina) and the Kapa Hifi HotStart Ready Mix (Kapa Biosystems) under the same PCR conditions mentioned above but with 8 cycles. After the indexed samples were purified (Agencourt AMPure XP beads, Beckman Coulter) they were pooled with all subsamples containing an equimolar amount of 16S rRNA gene amplicon. The quality of this library pool was then verified on the 2100 Bioanalyzer (Agilent Technologies, using the DNA High Sensitivity Chip). The amplicon libraries were sequenced by paired-end sequencing in a 2x300 bp run on the Illumina MiSeq platform (Illumina, St. Diego, USA).

***QIME, R, visualization.*** Sequences were processed using the Qiime2 environment (Bolyen et al., 2019). The filtering and merging of demultiplexed raw reads were performed using the dada2-plugin with default settings and removal of the primer sequences (Callahan et al., 2016). For taxonomic assignments the SILVA database release 138 (Quast et al., 2013) was pretrained with the respective primer pairs for Bacteria and Archaea (Pedregosa et al., 2011;Bokulich et al., 2018). The assignments were computed using the feature-classifier plugin (classify sklearn) with default settings and the pre-trained SILVA classifier (Bokulich et al., 2018). The phylogeny was calculated using the “align-to-tree-maffft-fasttree” pipeline and alpha diversity indices were calculated with the qiime diversity plugin using a sampling depth of 2053 for Bacteria and 2625 for Archaea (Price et al., 2010). Principle Coordinate Analysis (PCoA) based on unweighted UniFrac distances, MANOVA to calculate the significance of the differences in the unweighted UniFrac distances between RNA and DNA and the different sampling time points, and differential abundance analysis were performed in R: A language and environment for statistical computing. R Foundation for Statistical Computing, Vienna, Austria. URL https://www.R-project.org/ using the microeco package (Liu et al., 2021). Rarefacation for PCoA analyses was performed with different subsample sizes: (i) with the default sample size of 1000, (ii) with individual sample sizes of 2053 for Bacteria and 2625 for Archaea.

***Quantitative analyses: Cell numbers, CARD-FISH, qPCR and MPNs****.* For total cell counts via diamidino-2-phenylindole (DAPI) and CARD-FISH analyses sediment samples were fixed on board in 4% formaldehyde/PBS and incubated over night at 4°C. After washing the samples twice with PBS buffer they were stored at -20°C in PBS/EtOH [1:1] until further processing. Cells were then separated from the sediment by ultrasonication (2 times: 20%, 20 cycles, 20 seconds) and diluted with sterilized PBS [1:4000] before they were fixed on 0.2 µm polycarbonate filters by vacuum filtration (-200 mbar). The filters were then embedded in low gelling point agarose (0.2%) and dried for 30 min at 37°C. While DAPI staining was made for all depth horizons of MIC 1 of October 2018 and MIC 1 of March 2019, CARD-FISH analyses were performed only representatively for the surface/intermediate zone (samples 0-3 cm depth horizons) and the AOM zone (sample 20-22 cm depth horizon) from both of these MICs. CARD-FISH preparation was conducted following the standard protocol (Pernthaler et al., 2002) with minor modifications. Filter sections for permeabilization of bacterial and archaeal cells were incubated in lysozyme solution (10 mg ml-1, PanReac AppliChem, Darmstadt, Germany) at 37°C for 60 min and in proteinase k solution (15 µg ml^-1^, Sigma-Aldrich, Darmstadt, Germany) at 37°C for 15 min. Endogenous peroxidases were inactivated by incubating filters in 0.15% H_2_O_2_ in MeOH for 30min at room temperature. Hybridization took place at 46°C for 3 hours with a 300:1 hybridization mix (35% formamide for both, EUB338I-HRP and Arch915-HRP). For the amplification, filters were incubated at 46°C for 45min (7µl Alexa Fluor^TM^ 488, Invitrogen^TM^ by Thermo Fisher Scientific). Filters were counterstained with DAPI (10 min at room temperature) and embedded in Citifluor.

Relative abundances of Bacteria and Archaea via qPCR were estimated for the samples in 2-3 and 20-22 cm depth of the cores analyzed with DAPI. A total of 20 ng of cDNA synthesized as described in the section before was used as a template for amplifying the hypervariable V3/V4 regions of bacteria and the hypervariable V4/V5 regions of archaea of the 16S rRNA gene. Therefore, the same primer pairs that have been used for Illumina^®^ sequencing were used, but for qPCR the Illumina^®^ adaptor overhang nucleotide sequence was removed. The SYBR^®^ Select Master Mix, CFX (Applied Biosystems^®^ by Life Technologies^TM^) was used to set up the qPCR reaction. The amplification was performed with the CFX96^TM^ Real-Time System C1000 Touch Thermal Cycler (BioRad, Hercules, CA, USA) under the following conditions: 95°C for 2 minutes followed by 40 cycles of 98°C for 15 seconds, 59°C for 20 seconds, and 72°C for 30 seconds. Five technical replicates were measured and used to calculate relative quantitative abundances. Next to the samples a non-template control, a non-reverse transcriptase control, and a positive control was set up.

As we originally expected significant changes in the iron cycles across seasons, we used the Most Probable Number (MPN) method to estimate the number of microaerophilic and Fe(III)-reducing microbes (performing Fe(III) reduction coupled to organic carbon or dihydrogen oxidation). For the MPNs, artificial sea water media (ASW) was used containing the following salts per liter: 17.3 g NaCl, 8.6 g MgCl_2_·6H_2_O, 0.025 g MgSO_4_·7H_2_O, 0.99 g CaCl_2_·2H_2_O, 0.39 g KCl, 0.059 g KBr, 0.25 gNH_4_Cl, and 0.05 g KH_2_PO_4_. The medium was prepared anoxically with a headspace of N_2_/CO_2_ (80:20) in a Widdel flask and buffered with 22 mM bicarbonate buffer. For microaerophilic bacteria the bicarbonate buffer was lowered to 10 mM. pH was adjusted to pH 7 and 1 mL vitamin solution (Widdel and Pfennig, 1981), 1 mL selenite-tungsten solution (Widdel, 1980) and 1 mL trace element solution (Tschech and Pfennig, 1984) was added per liter. To inhibit sulfate reduction, 5 mM Na_2_MoO_4_ was added to the media.

For enrichment of Fe(III)-reducers, we added 5 mM ferrihydrite, 5 mM sodium acetate and 5 mM sodium lactate. Ferrihydrite synthesis was performed according to Raven et al. (1998) and Schwertmann and Cornell (2008).

Sediment for the MPNs was taken from the same core as the samples for 16S RNA sequencing. 1 g of homogenized sediment was mixed with 9 mL of ASW media and the solution was sequentially diluted tenfold (10-12 dilution steps). Samples from this dilution series were further aliquoted into each of the MPNs for specific metabolisms so that the results were directly comparable within each sample.

Quantification of Fe(III)-reducers with organics were set up under an N_2_ atmosphere in an anoxic chamber and conducted in 96 well deep-well plates. 100 µL of each dilution was mixed with 900 µL of the appropriate media in 7 replicate wells. The last row of the 96 well plate was left uninoculated and used as a sterile control. Therefore 7 replicates were performed with a sterile control. For anoxic incubation the deep well plates were placed in the Anaerocult system (Merck, Germany) and anoxic conditions were verified with an O_2_ indicator stick (Merck, Germany). MPNs for Fe(III)-reducers with hydrogen were conducted in triplicates in Hungate tubes with 9 mL of media and 1 mL of the dilution series. The headspace was flushed with a H_2_/CO_2_ mixture (10:90) twice per week.

Microaerophilic Fe(II)-oxidizer MPNs were cultivated in zero-valent iron (ZVI) plates (Oct 2018) and gradient tubes (March 2019), both times in triplicates. ZVI plates were conducted as described (Laufer et al., 2016). For the ZVI plates, ca. 60 mg ZVI powder (200 mesh; metal basis; Alfa Aesar, Ward Hill, MA) was placed into a petri dish with 7.2 mL ASW and 0.8 mL of sample from the dilution series. For each dilution step triplicates and a sterile control were set up. The petri dishes were placed in an acrylic gas-tight jar (Merck, Germany) with a gas pack to create a micro-oxic atmosphere (6 – 10 % atmospheric O_2_ concentration, BD GasPack EZ Campy;Becton, Dickinson and Co.,NJ).

MPN in gradient tubes were performed after Emerson and Moyer (1997). In these gel-stabilized tubes opposing gradients of Fe(II) and O_2_ are formed. The bottom layer contained 1 % (wt/vol) agarose solved in a mixture of FeS and ASW media (1:1) and was added to the tube first (0.75 mL). On top, 3.75 mL of ASW media with 0.15 % (wt/vol) agarose is added. The headspace is flushed with N_2_/CO_2_ (80:20) and the tubes are closed with a butyl stopper. The tubes are stored for 12 to 24 h before inoculation. During inoculation the butyl stopper is removed, 100 µL of the dilution series is added and the tube is closed again. Per dilution step samples were incubated in triplicates with a sterile control.

All MPNs were incubated for 6-8 weeks and Fe(II) oxidation and Fe(III) reduction was inferred from a colour change from colourless to brown (oxidation) or from brown to black (iron reduction). Growth of cells in ZVI plates was additionally verified with a fluorescence microscope following staining with DEAD/LIVE stain. Positive growth in gradient tubes was identified by the formation of a distinctive orange oxidation band. All positive wells/tubes were counted and the most probable number was calculated using the software Klee (Klee, 1993)

**Supplementary Results and Discussion**

**Water Column Biogeochemistry**

The increasing methane concentration gradients towards the seafloor (supplementary figure S1) indicate that the sediments are a source of dissolved methane to the overlying water column at certain times of the year. In relation to the sediment sampling campaign, bottom water methane concentrations were nearly five-fold higher in March (168 µM) than in October (35 µM) and are likely explained by the early spring bloom (Bange et al., 2010). Generally, bottom water methane concentrations appear to be up to 20% lower than those monitored for March and October roughly 10 years ago (Steinle et al., 2017). This coincides with a decline of primary production over time (Lennartz et al., 2014) and thus a lower C-flux to the sediments.

Northwestern Europe experienced a pronounced heatwave in summer 2018 (see, e.g. Kueh and Lin, 2020). This resulted in a temperature anomaly of about 3.5 °C in 1 m water depth at Boknis Eck in August 2018, which, however, was not visible in near bottom waters at 25 m depth during summer/autumn 2018 (Gindorf et al., 2022).

Nano- and pico-plankton cell counts varied considerably between the seasons (March and October) and between the tested years of 2015 to 2019 (supplementary figure 2A, B) with larger biomass of nano- and picoplankton in summer and autumn. For October 2017, 2018, and 2019 picoplankton, i.e. small cyanobacteria like *Synechococcus*, increased 8-fold (2017) and 35-fold (2018), relative to March of the same years, reaching the so far highest cell counts in October 2018 (supplementary figure S2B) just after the heatwave experienced in summer 2018. A shift from larger cells to smaller cells with decreasing nutrient concentrations and increasing temperatures has been suggested to impact trophic levels with zooplankton preferring larger cells (Lewandowska et al., 2014). However, this shift in cell sizes has not been shown to affect the carbon export to deeper layers (Lapoussière et al., 2013;Lomas et al., 2022).

Total dissolved amino acids (TDAA) can be used as an indicator of the degradation stage of OM. Mean concentrations of TDAAs between 2015 and 2019 were rising with the spring bloom with a second larger increase observed in summer and autumn (supplementary figure S2C) implying a higher OM turnover in the summer. Both the years 2018 and 2019 were at the higher end of measured concentrations with overall largest TDAA concentrations observed in late summer and fall in 2018. The fraction of TDAA of DOC in the water column, which can be used to trace the contribution of labile compounds to the DOM pool (Gaye et al., 2022 and references therein), is generally low at Boknis Eck due to a large anthropogenic background of mainly refractory terrestrial DOC. Still, the mean value of 1.86 % of TDAA in DOC in the water column in October of 2018 represents the largest relative contribution of TDAA to DOC in 2018 indicating a comparatively large OM turnover in summer and autumn of 2018 in the water column, following the heatwave. This is also true with respect to other years (1.04 – 1.54 % in October of 2016, 2017 and 2019) indicating a comparatively large OM turnover in summer and autumn of 2018 in the water column. The concentration of TDAA decreased in winter and increased in March 2019 but were generally lower than in fall.

**Sediment geochemistry and modeled rates**

Alkalinity is a measure for metabolic carbon release in anoxic sediments. While the maximum alkalinity observed during our two campaigns was similar (~25 meq l^-1^) (figure 1D, F). In October 2018 sediments at Boknis Eck were sulfidic throughout the cored depth interval. Hydrogen sulfide concentrations increased from 0.1 mM in the uppermost cm to ~4 mM at the lower end of the core. Sulfate concentrations decreased from 20 mM at the surface to <3 mM in the deepest pore water sample. The two cores retrieved in October differed in that sulfate concentrations plateaued at 6 to 12 cm sediment depth in MIC1 whereas sulfate concentrations decreased steadily in MIC2 (figure 1A, B, C and supplementary figure S9A, B). Dissolved Fe concentrations were low in all cores (figure 1A, B, C), which was related to precipitation of Fe sulfide minerals under sulfidic conditions (supplementary figure S3). Pore water Mn profiles were characterized by maxima at 3.5 cm (MIC1) and 6 cm (MIC2) and decreased below (figure 1A, B, C). Decreasing Mn concentrations with depth were likely related to precipitation of Mn carbonates.

Pore water chloride profiles in Boknis Eck differed during our sampling campaigns in October 2018 and March 2019 (figure 1D-F, supplementary figure S9). Since chloride is a conservative parameter, i.e. is not affected by biogeochemical processes, this down core chloride variability must be related to salinity variations in the bottom water. These would be transmitted to the pore water by diffusive or non-local transport processes such as bio-irrigation (for more discussion see below under modeled rates).

Sedimentary reactive Fe was dominantly present in the Fe_py_ and Fe(II)_HCl_ fractions whereas Fe_dith_ and especially Fe(III)_HCl_ were of subordinate importance (supplementary figure S3). During the October campaign, surface sediments (0-2 cm) were characterized by higher Fe concentrations in all reactive Fe pools. Otherwise, there was comparably little downcore variability in reactive Fe speciation. During the October campaign, subsurface sediments were characterized by detectable concentrations of Fe(III)_HCl_ between 10 cm and the lower end of the core whereas during the March campaign Fe(III)HCl was not detectable in subsurface sediments. The average extent to which Fe oxide minerals have been converted to Fe sulfide minerals was identical during the two sampling campaigns (October: 0.73 ± 0.04, n = 13, March: 0.75 ± 0.02, n = 14).

***Temperature and dissolved chloride.*** The model is first run assuming that bio-irrigation does not contribute to the transport of solutes (a = 0) such that the transport of heat and dissolved tracers is largely dominated by heat conduction and molecular diffusion, respectively. Since thermal conduction is much faster than molecular diffusion, changes in bottom water temperature affect the entire model column while only the upper section is affected by changes in bottom water salinity (Supplementary figure S13).

Dissolved chloride concentrations calculated for October 23^rd^ 2018 and March 15^th^ 2019 are compared to pore water data taken on these dates to validate whether the transport of solutes was dominated by molecular diffusion or significantly affected by other processes. The model generally achieved a good fit for both dates (supplementary figure S9, S10). One of the two sediment cores taken in October 2018 (MIC2) showed a strong chloride depletion at 14–20 cm sediment depth that was not reproduced by the model (figure 1D-F, supplementary figure S8). This depletion was not observed in the other core (MIC1) taken at the same date and location. The difference between the two cores suggests a strong small-scale spatial variability. MIC2 was probably affected by a process that transported chloride-depleted bottom water towards the base of the core during April to May 2018 when bottom waters were depleted in chloride and enriched in oxygen (supplementary figure S1). It is likely that MIC2 was affected by additional processes that transported chloride-depleted bottom water towards the base of the core. It is likely that this transport occurred during April – May 2018 when bottom waters were depleted in chloride (supplementary figure S15). The distribution of reactive tracers was generally well reproduced by the model (supplementary figure S10). A significant deviation between model and data was also observed for dissolved sulfate and sulfide in MIC1 whereas sulfate in MIC2 was consistent with the model (no sulfide data available for MIC2). This is an interesting observation since the model deviates from dissolved chloride measured in MIC2 but is consistent with the data recorded in MIC1. It seems that rapid microbial processes restored the diffusion-controlled sulfate profile in MIC2.

***Model limitations.*** In our model, rates change over time due to changes in sediment temperature. It should, however, be noted that we did not consider seasonal changes in the rain rate of POC and in the oxygenation of bottom waters that affect microbial rates especially in the surface layer (Dale et al., 2013). Hence, we are not able to fully resolve the temporal variability of turnover rates in Boknis Eck sediments.

Moreover, we made no attempt to resolve the many overlapping processes occurring in the oxic – suboxic surface layer. These include oxygen consumption, denitrification, DNRA (dissimilatory nitrate reduction to ammonium), ANAMMOX (anaerobic ammonium oxidation), manganese and iron reduction driven by organic matter degradation and sulfide oxidation. We lumped all these processes into the two reactions: R_O_ representing POC degradation pathways other than sulfate reduction and methanogenesis and R_SO_ representing sulfide oxidation using oxygen, nitrite, nitrate, manganese, iron as electron acceptors. We used this strategy because we do not have the data that are needed to resolve the various processes occurring in the surface layer.

**Microbiology**

***Abundance and phylogenetic diversity of the microbial communities.*** Boknis Eck sediment cell numbers (figure 2A) are in the upper range of what is commonly found in organic-rich marine sediments at these sediment depths (cf. Schippers et al., 2012). The proportion of active Bacteria, as indicated by CARD-FISH analyses relative to DAPI stained cells, in the surface sediment is in the lower range of previous observations but deeper down corresponds with what has been found in marine sediments before (figure 2B) (Ishii et al., 2004).

The drop of cell numbers with depth is not reflected in the Shannon diversity index (SI) (supplementary figure S16). The SI considers both the number of species (richness) and their relative abundance (evenness). The SIs for the bacterial and archaeal communities are overall high (Jochum et al., 2017;Otte et al., 2018;von Hoyningen-Huene et al., 2022). The higher SI for DNA- than for RNA-based analyses is also found in other marine sediments (Otte et al., 2018;von Hoyningen-Huene et al., 2022). DNA analysis includes dead cells and extracellular DNA (eDNA). Except for the SI for active Archaea declining with depth in MIC1 from fall 2018, SIs of Bacteria and Archaea from all other sediment cores demonstrate no considerable up- or downwards trend with depth (supplementary figure S16).

The generally high phylogenetic diversity at Boknis Eck is also reflected in the large number of low abundance (<3%) Bacteria in all depth horizons, ranging from at least 18% to 81% (median 46%) (supplementary figures S6) and may mark a vital component for community dynamics. The sediments exhibit a very large proportion of low abundance Bacteria and Archaea (supplementary figures S6, S7). These findings are in line with other studies on 16S tags in marine sediments (Adam et al., 2019 and references therein). Since most of the microbes found in marine sediments are uncultured taxa and their physiology remains enigmatic (Lloyd et al., 2018), a tremendous catalytic potential remains hidden among the microbial dark matter in seafloor habitats (Zamkovaya et al., 2021).

***Vertical zonation of active prokaryotes in sediments and their potential metabolisms.*** Based on 16S tags from transcripts in zone 1 *Beggiatoaceae* and *Nitrosopumilus* are typically the prevailing Bacteria and Archaea, respectively (supplementary figures S6A-D, S7A, B). The former are found in sulfidic environments, such as sediments (Winogradsky, 1887;Jørgensen, 1977). The latter are prominent representatives in the oceanic water column and in sediments (Kerou et al., 2021). In zone 1 and 2 a mix of organotrophic Bacteroidota and Pirellulales, sulfate-reducing Bacteria (SRB) (primarily SRB-SEEP1 of the *Desulfobacteria*) and a phylogenetically diverse mix of potential sulfide-oxidizing (autotrophic) Bacteria are found (supplementary figure S6A-D). The latter are mostly affiliated with uncultured *Thiotrichaceae* and *Sulfurimonaceae*. In the deepest studied zone (zone 3) the organotrophic Bacteroidota and some *Pirellulales* lineages remain but are accompanied by *Aminicenantes* of the candidate phylum OP8, likely with a fermentative saccharolytic lifestyle (Kadnikov et al., 2019 ), JS1 lineage included in the candidate phylum Atribacteria (OP9), which are expected to be anaerobes and involved in primary fermentation of carbohydrates or secondary fermentation of organic acids producing H_2_, CO_2_, acetate, and formate (Nobu et al., 2016;Lee et al., 2018). Surprisingly *Poribacteria* are found in the deeper zone - although they are typically associated with sponges – and are described as having an aerobic mixotrophic lifestyle, i.e. oxidative phosphorylation, glycolysis and autotrophic CO_2_ fixation (Kamke et al., 2013).

Among the Archaea (supplementary figure S7A, B), the versatile metabolisms of the anaerobic Bathyarchaeia include thiosulfate oxidation, acetogenesis, methane metabolism, as well as dissimilatory nitrite and sulfate reduction (Zhou et al., 2018). They can utilize detrital proteins, polymeric carbohydrates, fatty acids, aromatic compounds, methane (or short chain alkane) and methylated compounds. Their flexible substrate spectrum likely makes them abundant throughout Boknis Eck sediment cores but also throughout other sediments. Several potential interactions with ANME, acetoclastic methanogens and heterotrophic bacteria are suggested (Zhou et al. 2018). *Methanofastidiosa* likely operate hydrogen–dependent methylotrophic methanogenesis (Zhang et al., 2020) and appear displaced by *Nitrosopumilus* and ANME. Metagenomic assessments from coastal sediments have indicated a significant non-random association of these and other methanogens with Woesearchaeota, acetate degrading sulfate-reducers and sulfur-cycling Campylobacterales (to which *Sulfurimonas* belong) (Zhang et al., 2020). All of which are detected in Boknis Eck sediments above zone 3 (supplementary figure S7A, B). The Woesearchaeota are in all Boknis Eck sediment cores in much lower numbers, with highest numbers in the surface regions declining with increasing depth. As for most of the here mentioned prokaryotes, physiological traits are derived from MAGs (metagenome assembled genomes) and suggest an anerobic fermentative lifestyle (cf. Huang et al., 2021). Lokiarchaeota make up >20% of archaeal 16S tags in most depth horizons and are known to catalyze acetogenesis, fermentation and hydrogen-dependent carbon fixation (Spang et al., 2015;Orsi et al., 2020). Co-occurrence analyses indicate a significant non-random association of Lokiarchaeota with members of the Marine Benthic Group D (MBG-D) (Zhou et al., 2019). Genomic sequencing has suggested that MBG-D can transport and assimilate peptides and generate acetate and ethanol through fermentation. Additionally, these organisms appear to encode for two autotrophic CO_2_ fixation pathways (Zhou et al. 2018).

***Allocating estimated metabolic processes to phylotypes.*** Bioturbation is a process whereby fresh organic matter can be introduced into deeper sediments. For Boknis Eck sediments bioturbation has been documented down to 15 cm (Dale et al. 2013). This biological activity is known to support an elevated phylogenetic diversity among SRB (Jochum et al., 2017). This is also the case in the Boknis Eck sediments, where elevated diversity among SRB in top sediments coincides with modeled peaks in SRR (figure 3A, B).

Sulfide-oxidizing, nitrate reducing *Beggiatoaceae* appear common in Boknis Eck sediments at certain times of the year and were detected in samples collected in March and June 2002, January 2003 and during summer/hypoxia 2010 (Preisler et al., 2007;Dale et al., 2013). However, no genetic analyses were conducted with these samples to confirm that mats were indeed dominated by *Beggiatoaceae*. These sediments with apparently large proportions of *Beggiatoaceae* biomass, measured SOR that were in the same order of magnitude as SOR estimated for 2018 with hardly any detectable *Beggiatoaceae* (4%). Detailed investigations on vertical *Beggiatoaceae* distributions in 2002/2003 sediment cores showed that they were dominantly in the upper 2 cm and sulfide only started accumulating below this depth horizon (in most cores less than 0.5 mM sulfide at 3 cm depth), the onset accompanied by a peak in sulfide oxidation rates (Preisler et al., 2007). Deeper profiles indicate that sulfide accumulation (>1 mM) started at 10 cm depth (Preisler et al., 2007). Coinciding with this trend is the lack of sulfide accumulation in the upper 10 cm during hypoxia 2010 with potential *Beggiatoaceae* dominating the upper sediments (Dale et al., 2013). In line with these previous observations, we observed sulfide enrichment in our samples only below sediment horizons where *Beggiatoaceae* were enriched (1.3 mM at 5.5 cm) (MIC1 spring 2019) (figure 1C). In contrast, in the autumn 2018 MIC1 core, where *Beggiatoaceae* were hardly detectable, sulfide already reached levels of 1.9 mM at 2.5 cm depth (figure 3A). Preisler and colleagues (2007) argued that the discrepancy between the total SOR and the oxidation catalyzed by *Beggiatoaceae* is due to abiotic sulfide oxidation coupled to FeOOH reduction (Preisler et al., 2007). Intriguingly, in March 2019 where *Beggiatoaceae* were dominant, dissolved Fe spiked in the top sediments, suggesting that sulfide oxidation with FeOOH was possible. In contrast, we did not see this spike of dissolved Fe in the investigated October 2018 core where *Beggiatoaceae* were hardly noticeable. In March 2019, *Beggiatoaceae* sulfide oxidation activity contributes to controlling sulfide availability, thereby possibly indirectly affecting iron-sulfide precipitation in the sediments and the capability of iron-minerals (e.g., FeOOH) to react with sulfide and thus other molecules (e.g. phosphate). A clear shift from *Beggiatoaceae* to phylogenetically diverse sulfide oxidizing Bacteria is visible for the different seasons. The large proportion of uncultured *Thiotrichacea* is pronounced in the intermediate zone. Related cultured representatives are demonstrated to oxidize sulfide and fix carbon autotrophically.

A shift among the SRB affiliated predominantly with SEEP-SRB1 in zone 2, to *Desulfatiglans* (both SEEP-SRB1 and *Desulfatiglans* belong to the family *Desulfobacteraceae*) prevailing in zone 3 is noted (supplemental figure S6A-D). *Desulfatiglans* are typically described as aromatic compound degrading dissimilatory sulfate reducers (Suzuki et al., 2014). *Desulfatiglans* has been documented to co-occur with ANME-1b (Kevorkian et al., 2021). At Boknis Eck they appear with both ANME-1b and ANME-2b in significant numbers (up to 45%) (supplementary figure S7A, B), with ANME most likely being responsible for peaks of modeled AOM (anaerobic methane oxidation) rates in zone 3 (figure 3C, D). Yet, modeled SRR in the AOM peaking zone is negligible with 0.58 and 0.15 µmol S cm^-3^ yr^-1^. Since sulfate levels drop from ~20 mM in the surface to <4 mM in zone 3 (figure 1), (some of) these SRB may revert to alternative metabolic processes and *Desulfatiglans* strain heterogeneity may enable multiple phenotypes to persist. More recent work has illustrated that some members of this group appear to lack genes known to encode SR and instead have genes for acetogenesis and fermentation (Jochum et al., 2018) giving them an advantage over SRB.

Modeled methanogenesis rates for October 2018 and March 2019 are shallower (supplementary figure S4C, D) than what has been measured in other years in the same seasons (Maltby et al., 2018) and peaked at 0-2 cm (October 2018) and at 2.5 cm (March 2019). These peaks co-occur with those modeled for SR. Although experiments have suggested that methylotrophic compounds as alternative non-competitive substrates enable methanogenesis to occur in the SR zone (Oremland and Polcin, 1982;Maltby et al., 2018), the coexistence of SR and methanogenesis with shared substrates without sulfate depletion has also been demonstrated before (Sela-Adler et al., 2017). In the last years the phylogeny of methanogens exclusively related to Euryarchaota (Garcia et al., 2000), has been expanded to include other phyla such as Candidatus Bathyarchaeota (Evans et al., 2015) and Thermoplasmata (Poulsen et al., 2013). Representatives of both these groups are found in Boknis Eck sediments.

Aerobic methanotrophs only make up less than 4% of the bacterial 16S tags of transcripts (data not shown). In contrast, ANME - known to catalyze AOM - make up a large proportion of the Archaea in October 2018 sediments in zone 3 (up to ca. 45% of the archaeal 16S tags of transcripts) (supplementary figure S7A, B). The deepest sediment horizon collected in March 2019 sediment samples was at 22-24 cm, where ANME-1b made up ca. 20% of Archaea. The estimated AOM rate peaks correspond with peaks in ANME-1b 16S tags, which appear to be at greater depth horizons in the March 2019 sediments (figure 3C, D).

To estimate the per cell rates, we deduced the likely number of active cells putatively performing sulfide oxidation, SR and anaerobic methane oxidation from the proportion of bacterial or archaeal 16S tags from transcripts that are affiliated with potential sulfide oxidizing Bacteria (SOB), sulfate-reducing Bacteria (SRB) and ANME and the relative abundance of active Bacteria or Archaea, respectively, as indicated by CARD-FISH. For SOB we used data from 0-1 cm and 1-2 cm, and for SRB and ANME from 2-3 cm and 20-22 cm for October 2018 and the March 2019 samples.

The estimated per cell SRR for the surface sediments in October 2018 were 0.2 fmol cell^-1^ d^-1^ and were 5.5-fold higher than those from surficial sediments in March 2019. The calculated per cell SRR in the 20-22 cm layer are up to two magnitudes lower and those from 2018 are 2.2-fold higher than those from March 2019 (supplementary figure S14). The per cell AOM rates were more stable over time, where estimated rates for surface near sediments are around 0.4 fmol cell^-1^ d^-1^ and drop by 15% and 30% for October 2018 and March 2019, respectively. Our estimated per cell rates are in the range of what has been calculated before for the environment: per cell SRR in the environment are typically on the order of 10^-2^ to 10 fmol sulfate reduced cell^-1^ d^-1^ and per cell rates of AOM are commonly in the range of 10^-4^ to 10 fmol methane oxidized cell^-1^ d^-1^  (Orcutt and Meile, 2008 and references therein).

In line with higher modeled rates for October 2018, modeled SOR are three-fold higher for October 2018 sediments (supplementary figure S5A, B), corresponding with higher general cell numbers and more active cells in the upper sediments (figure 2). Cell numbers and active cells in October are 20% higher than those from March 2019 and the per cell SOR are 20-fold higher for the October 2018 top 0-1 cm sediments (supplementary figure S14); although in the upper region where SOR peak in October 2018 15% and 20% of the 16S rRNA gene tags of transcripts are assigned to potential sulfide oxidizers, while in the March 2019 surface sediments 55% and 72% are associated with putative sulfide oxidizing Bacteria - 19% and 46% are of *Beggiatoaceae* -.

***Past and present microbial communities and changes to environmental conditions.*** According to Multivariate analysis of variance (MANOVA), DNA and RNA-based sequencing of bacterial and archaeal 16S tags demonstrate significant (p-value 0.001) differences between the total (dead and alive) (DNA) and active/dormant (RNA) parts of the communities of all tested sediment cores from October 2018 and March 2019. This is also visualized in the principal coordinate analyses (PCoA) (supplementary figure S17). This discrepancy between DNA and RNA profiling has been recognized for other habitats as well (Otte et al., 2018;Li et al., 2020;von Hoyningen-Huene et al., 2022) and can be explained by the difference in stability of DNA and RNA and what their detection reflects. DNA-based profiling targets living and dead cells as well as extracellular DNA (eDNA), mirroring the present (DNA+RNA) and past (DNA) communities (Torti et al., 2015). Under favorable environmental conditions, DNA from dead cells can persist up to weeks, months, and many years (even millennia) in marine sediments (Torti et al., 2015). In contrast, RNA in the environment is short lived (degradation starts within minutes) and 16S tag analyses of transcripts identifies only currently living (metabolically active and dormant) cells but excludes dead cells and eDNA (Blazewicz et al., 2013;De Vrieze et al., 2018).

***Spatial variability.*** On a spatial level, no significant differences are observed between the two cores collected in October 2018 and the two cores collected in March 2019 according to 16S tags from Bacteria and Archaea based on DNA-profiling and of Bacteria based on RNA-profiling. This suggests that the genetic fingerprint is relatively conform over space at the same times of the years. For RNA-based profiling, no spatial heterogeneity for Archaea can be resolved because only one core for each season is analyzed. Nevertheless, porewater chemistry suggests small-scale spatial heterogeneity (supplementary figures S9, S10). Possibly, the high number of low abundance taxa may mask significant shifts due to limitations of resolution levels of the analyses.

***Temporal variability.*** In contrast to the lack of detecting microbial spatial dynamics, significant temporal variability is noted for bacterial and archaeal 16S tags based on DNA profiling for October 2018 and March 2019 (p-value 0.001). These temporal dynamics are only reflected in the archaeal RNA (p-value 0.001) but not in the bacterial RNA. Possible explanations could be the time that the communities of the domains need to respond to environmental changes. The archaeal RNA patterns from October 2018 may still reflect the warmer, hypoxia conditions observed in September 2018, thus being different to the March 2019 community with colder, fully oxic bottom waters. On the contrary, the bacterial RNA patterns may already show some responses of the community to increasing oxygen levels in the bottom waters detected in October 2018 and thus the differences to the community from March 2019 with colder, oxic bottom waters is not pronounced. It needs to be kept in mind though, that the analysis is done for the entire core and therefore fine scale vertical variations for the different depth horizons are not included but only overall changes in the entire core community.

The significant levels of temporal variance (p-value 0.001) detected for bacterial and archaeal communities according to 16S tag analyses of DNA, i.e. present and past, is also apparent in the PCoA plots but only when assessing the maximum number of sequences, we can compare without losing subsamples during analyses (supplementary figure S17). They form two major groups in line with the two sampled years. Intriguingly, while samples from 2019 form a distinct group, those sediment horizons from 2018 are considerably more spread out than those from 2019. We posit that the 2018 DNA may be reflecting different stages of the present and past communities under colder, oxic versus warmer, hypoxic or anoxic bottom water conditions. When defining a smaller subset to be analyzed, DNA-based communities cluster (supplementary figure S17A). This could be related to the randomization process, where by chance more abundant sequences are likely favored over less abundant sequences when chosen for analyses. Hence, when by chance more low abundance sequences (cut-off value here 5%) are incorporated into the analyses a distinction between the seasons and years can be noted based on DNA (supplementary figure S17). The disproportionally large relevance of low abundant microbial lineages has been stressed before and concepts have suggested their major role despite relative minor abundance for shaping the community structure and affecting its functionality (de Cena et al., 2021).

***Origin of DNA.*** It currently remains uncertain whether DNA enrichments of specific 16S tags result from (i) entrainment through water column as a transport vehicle from sediments of other regions, (ii) are relicts of the last weeks or months or (iii) relicts from years ago. The first appears unlikely, since it would take many dead cells transported with a current to be deposited at different locations over time at Boknis Eck. More likely is that the DNA enrichment indicates past communities for which other environmental conditions were favorable. If these were directly from October 2018 and March 2019, we would expect to see that in the seasonal variation of the RNA profiling patterns but this is not the case. Hence, the changes must have happened further back in time, and we posit that at least some of the documented changes from October 2018 over time as reflected in DNA profiling could be dated back to possibly even years. The noted significant shifts in DNA for Bacteria and Archaea between October 2018 and March 2019 may reflect long-term changes manifested in DNA that can be stable over longer time periods (Torti et al., 2015). Nevertheless, only monthly sampling with combined porewater geochemistry and respective 16S tag analyses will shed light on these uncertainties.

***Most Probable Numbers of iron-metabolizing bacteria*** Micro-organisms were successfully enriched in MPN set ups for Fe(III)-reducers, both with acetate/lactate or dihydrogen as an electron donor. Enrichment was also successful in ZVI plates and gradient tubes which promote microaerophilic Fe(II)-oxidizing metabolisms.

Most probable number estimates of Fe(III)-reducers (supplementary figure S8) showed higher maximum abundances with both electron donors during the autumn 2018 cruise, however these differences were within the error range for most depths. There was also no clear trend in cell abundance with depth for either electron donor or sampling campaign, except for a generally decreasing trend in the C-utilizing Fe(III)-reducers from the spring 2019 cruise.

Cell numbers in the enrichments promoting microaerophilic, Fe(II)-oxidizing conditions showed the highest cell numbers of any of the set ups. In 2018, these range from 10² to 10^8^ cells/g sediment. We considered these numbers to be particularly high and were concerned about the specificity of the assay for microbes of this metabolism as the oxidation of ZVI can produce hydrogen as a by-product and would, therefore, also enable growth of hydrogen oxidizing bacteria. Therefore in 2019 we adopted the classical gradient tube cultivation for Fe(II)-oxidizers in which the Fe(II)-oxidizing microbes form a distinctive orange band during Fe(II)-oxidation. In this set up we found the numbers to be even higher at most depth intervals than determined in 2018 with the ZVI method (ranging from 10^4^ to 10^9^ cells/g sediment). Visual observation of the gradient tubes indicated that other metabolic types could also grow in the gradient tubes as the low dilution samples exhibited the formation of black mineral deposits (likely FeS minerals) during incubation (supplementary figure S12).

**Data Availability Statement**

Sequence data was deposited at the SRA (Sequence Read Archive) of NCBI (National Center for Biotechnology Information) under the BioProject ID PRJNA901661.

**Supplementary tables**

**Supplementary table S1:** Overview of analyses performed with the seven sediment cores of the 2018 and 2019 sampling campaigns. Bacteria and Archaea refers to the analysis of 16S gene tags. Geochemical analyses were performed with sediment cores taken adjacent to the microbiology cores.

|  |  | **October 2018** | | | | **March 2019** | | |
| --- | --- | --- | --- | --- | --- | --- | --- | --- |
|  |  | MIC1-M (D1) | MIC1-G (D1) | MIC3-M (D2) | MIC2-G (D2) | MIC1-M (D1) | MIC1-G (D1) | MIC3-M (D2) |
|  | Coordinates | 54°31.56’N,  10°02,52’E | | 54°31.60’N,  10°02,44’E | | 54°31.54’N,  10°02,57’E | | 54°31.73’N,  10°02,21’E |
| Bacteria | DNA seq | + | - | + | - | + | - | + |
|  | RNA seq | + | - | + | - | + | - | + |
|  | qPCR | 2-3 cm  20-22 cm | - | - | - | 2-3 cm  20-22 cm | - | - |
|  | CARD-FISH | 0-3 cm  20-22 cm | - | - | - | 0-3 cm  20-22 cm | - | - |
|  | DAPI | all horizons | - | - | - | all horizons | - | - |
| Archaea | DNA seq | + | - | + | - | + | - | + |
|  | RNA seq | + | - | - | - | + | - | - |
|  | qPCR | 2-3 cm  20-22 cm | - | - | - | 2-3 cm  20-22 cm | - | - |
|  | CARD-FISH | 2-3 cm  20-22 cm | - | - | - | 2-3 cm  20-22 cm | - | - |
| Pore water chemistry | | - | + | - | + | - | + | - |
| Fe speciation | | - | + | - | - | - | + | - |
| Modeled rates | | - | + | - | + | - | + | - |

MIC=minicorer, where D abbreviates Deployment, M stands for Microbiology analyses and G denotes Geochemistry; +: analysis was performed, -: analysis was not performed for the respective sample

**Supplementary table S2.** Depth-integrated rates (0 - 100 cm) for Oct. 23^th^, 2018 and March 15^th^, 2019 (in µmol cm^-2^ yr^-1^)

|  | *Oct. 2018* | *March 2019* |
| --- | --- | --- |
| *R_POC_ (µmol C cm^-2^ yr^-1^)* | *1460* | *494* |
| *R_O_ (µmol C cm^-2^ yr^-1^)* | *700* | *386* |
| *R_SR_ (µmol S cm^-2^ yr^-1^)* | *418* | *58.4* |
| *R_M_ (µmol CH_4_ cm^-2^ yr^-1^)* | *4.77* | *1.38* |
| *R_SO_ (µmol S cm^-2^ yr^-1^)* | *181* | *82.9* |
| *R_AOM_ (µmol CH_4_ cm^-2^ yr^-1^)* | *74.4* | *50.1* |

**Supplementary table S3.** Reactions and functions considered in the model. All rates are calculated in µmol cm^-3^ yr^-1^ where cm^3^ refers to the porewater volume.

| **Reaction** | **Kinetic rate law** |
| --- | --- |
| POC degradation | $R_{POC}=1000 \frac{B_{0}}{P}\left( x +B_{1} \right)^{B_{2}} \frac{T}{T_{mean}}$ |
| POC degradation in oxic/suboxic surface layer | $R_{O}=f_{O} R_{POC}$ |
| Dissimilatory sulfate reduction | $R_{SR}=\left( 1 - f_{O} \right) \frac{C_{{SO}_{4}}}{C_{{SO}_{4}}+K_{{SO}_{4}}}R_{POC}$ |
| Methanogenesis | $R_{M}=\left( 1 - f_{O} \right) \frac{K_{{SO}_{4}}}{C_{{SO}_{4}}+K_{{SO}_{4}}} R_{POC}$ |
| Sulfide oxidation | $R_{SO}=f_{O} k_{SO} C_{{TH}_{2}S}\frac{T}{T_{mean}}$ |
| Anaerobic methane oxidation | $R_{AOM}=k_{AOM} C_{{SO}_{4}}C_{{CH}_{4}}\frac{T}{T_{mean}}$ |
| Boltzmann function | $f_{O}=\frac{1}{1+e^{\frac{x - \frac{T_{mean}}{T_{BW}}}{0.3}}}$ |
| Porosity | $P=\left( P_{0} - P_{F} \right) e^{- \frac{x}{p}} + P_{F}$ |
| Burial velocity of porewater | $v_{W}=v_{f} \frac{P_{F}}{P}$ |
| Burial velocity of sediment | $v_{S}=v_{f} \frac{{1 - P}_{F}}{1 - P}$ |
| Heat capacity of bulk sediment | $H_{B}={H_{W} P + H}_{S} \left( 1 - P \right)$ |
| Thermal conductivity of bulk sediment | $\lambda_{B}={\lambda_{W} P + \lambda}_{S} \left( 1 - P \right)$ |
| Tortuosity | ${TO}^{2}=1 - 2 ln (P)$ |
| Diffusion coefficient in sediments | $D_{S}=\frac{D_{M}}{{TO}^{2}}$ |
| Molecular diffusion coefficients^1^ | $D_{M}=f (T, S)$ |

1: Molecular diffusion coefficients are calculated as function of temperature (T) and salinity (S) for each tracer using equations given in Boudreau (Boudreau, 1997).

**Supplementary table S4.** Parameter values applied in the model

| **Parameter** | **Symbol** | **Value** | **Units** |
| --- | --- | --- | --- |
| POC rain rate | RR_POC_ | 0.8 | mmol cm^-2^ yr^-1^ |
| Rate constant for R_POC_ | B_0_ | 0.5 | mmol cm^-3^ yr^-1^ |
| Rate constant for R_POC_ | B_1_ | 0.58 (calculated from RR_POC_) | cm |
| Rate constant for R_POC_ | B_2_ | -2.21 (calculated from RR_POC_) | - |
| Mean temperature | T_mean_ | 7.31 | °C |
| Monod constant for R_SR_ | $K_{{SO}_{4}}$ | 0.01 | mM |
| Kinetic constant for R_SO_ | k_SO_ | 500 | yr^-1^ |
| Kinetic constant for R_AOM_ | k_AOM_ | 5 | mM^-1^ yr^-1^ |
| Porosity at x = 0 | P_0_ | 0.95 | - |
| Porosity after compaction | P_F_ | 0.85 | - |
| Attenuation coefficient for P | p | 3 | cm |
| Burial velocity after compaction | v_f_ | 0.39 | cm yr^-1^ |
| Heat capacity of porewater | H_W_ | 4.14 | J cm^-3^ K^-1^ |
| Heat capacity of solids | H_S_ | 2.15 | J cm^-3^ K^-1^ |
| Thermal conductivity of porewater | λ_W_ | 0.58 | W m^-1^ K^-1^ |
| Thermal conductivity of solids | λ_S_ | 2.5 | W m^-1^ K^-1^ |

**Supplementary table S5.** Rate term applied in partial differential equation Eq. 2 for each species

| **Species** | **Rate term (R)** |
| --- | --- |
| Chloride | 0 |
| Sodium | 0 |
| Sulfate | - 59/106 R_SR_ – R_AOM_ + R_SO_ |
| Methane | 59/106 R_M_ – R_AOM_ |
| Sulfide | 59/106 R_SR_ + R_AOM_ - R_SO_ |

**Supplementary figures**

**Supplementary figure S1.**


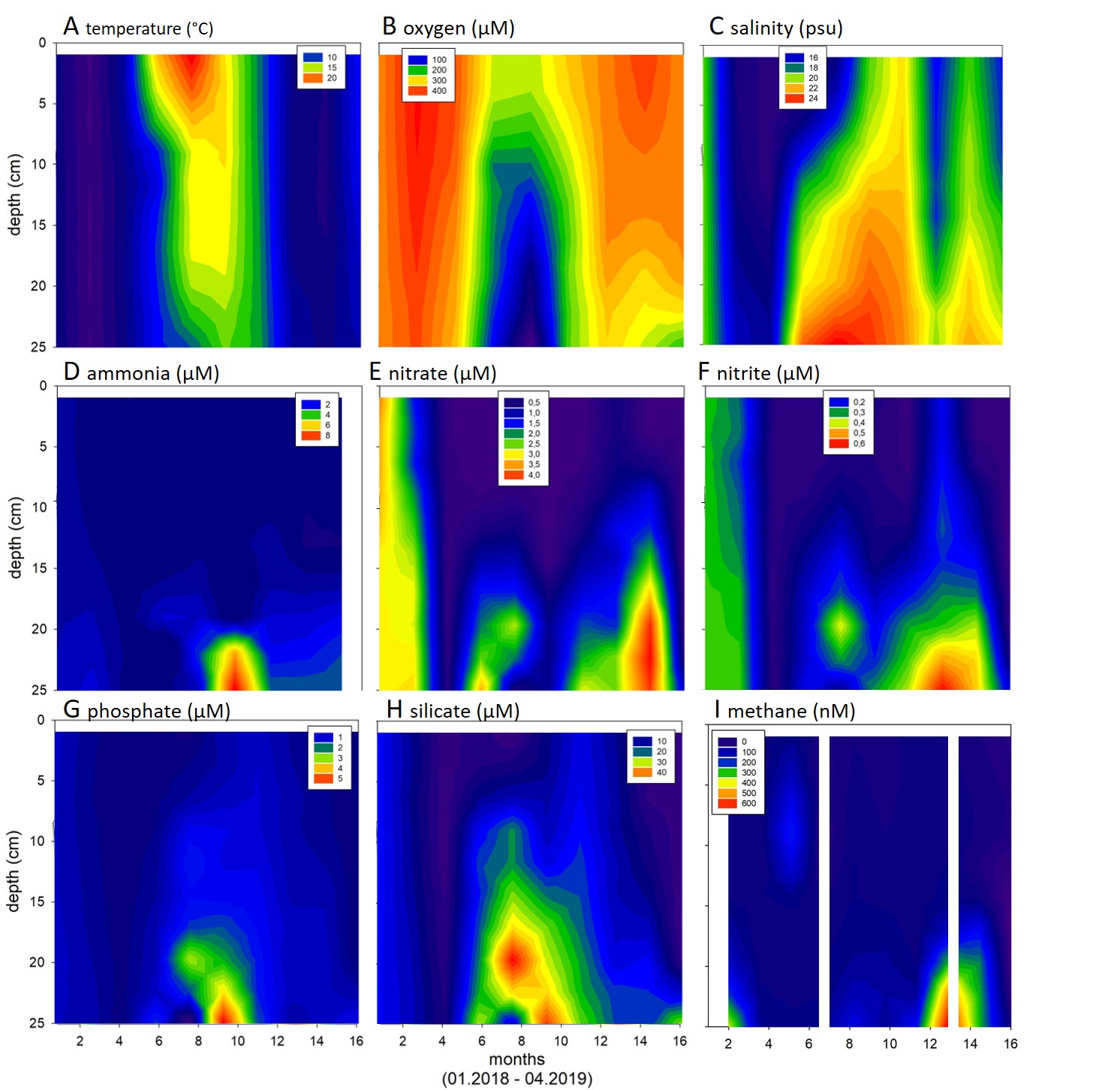


**Seasonal variability of the water column parameters.** (A) temperature (°C), (B) oxygen (µM), (C) salinity (psu), (D) ammonia (µM), (E) nitrate (µM), (F) nitrite (µM), (G) phosphate (µM), (H) silicate (µM), and (I) methane (nM) at Boknis Eck from January 2018 to April 2019. Our sediment sampling campaign took place in October 2018 and March 2019.

**Supplementary figure S2.**

**
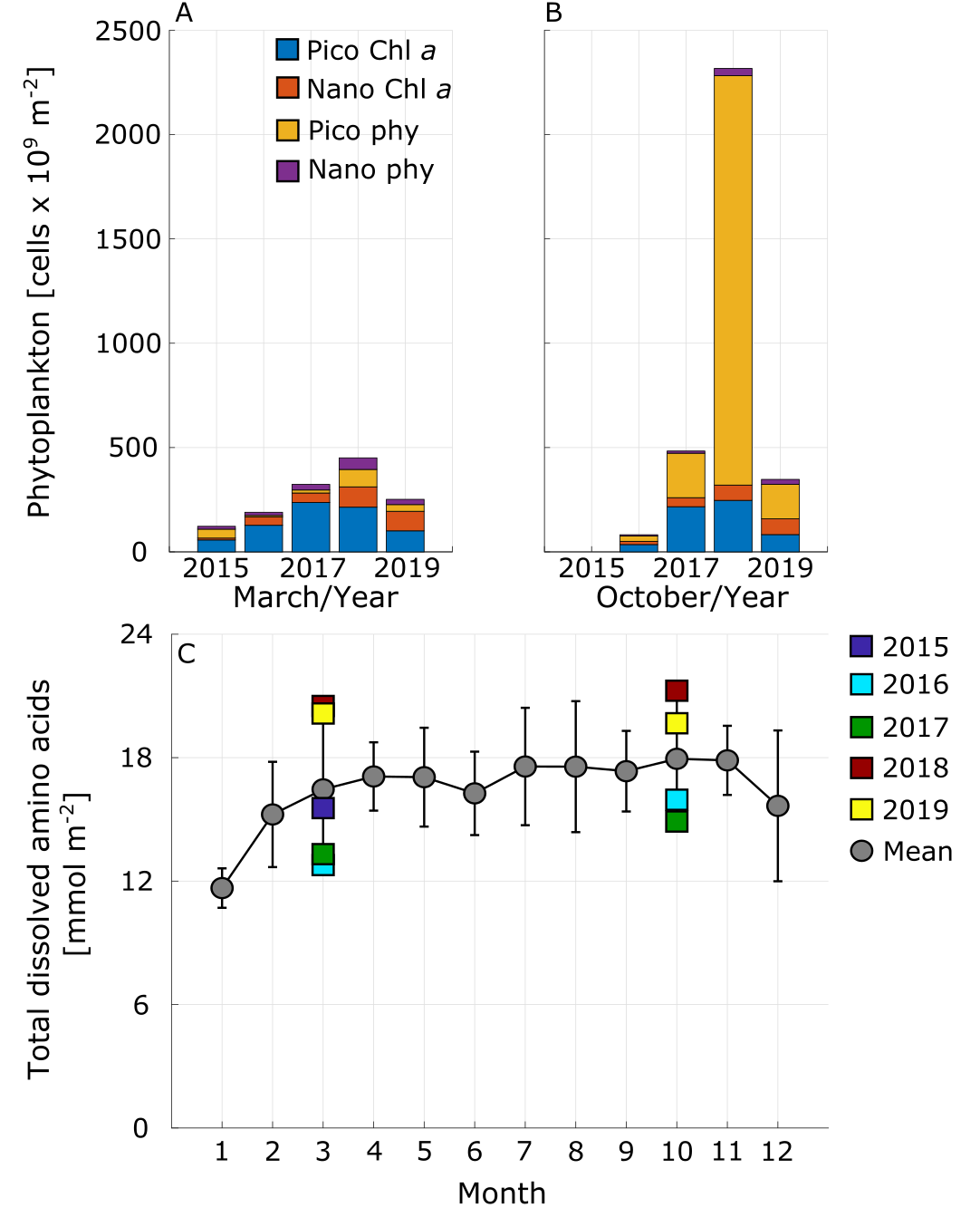
**

**Phytoplankton (A, B) and total dissolved amino acids (C) for the water column.** Phytoplankton in March (A) and October (B) between 2015 and 2019 and mean and standard deviations of total dissolved amino acids for the water column for the years 2015 to 2019 and specifically for the months March and October in C. Phytoplankton cell counts were determined according to their fluorescence either based on the pigments chlorophyll *a* (Chl *a*, red fluorescence, including e.g. small diatoms) or phycoerythrin (phy, orange fluorescence, including cyanobacteria). Furthermore, they were divided into pico- (< 2 µm) and nanoplankton (2 – 20 µm).

**Supplementary figure S3.**


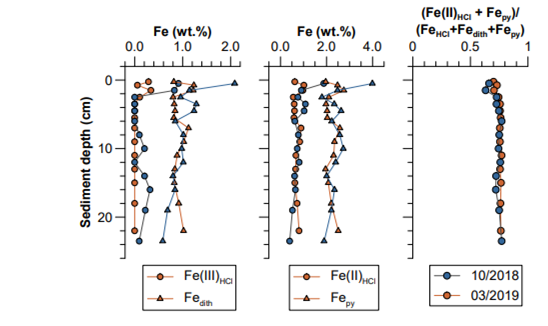


**Profiles of biogeochemically reactive Fe species in the sediment solid phase in October 2018 (blue) and March 2019 (red).** (A) poorly crystalline (Fe(III)_HCl_) and crystalline (Fe_Dith_) Fe oxides; (B) Fe(II) monosulfides and pyrite; (C) extent to which solid phase Fe(III) has been converted to solid phase Fe(II) (1 equals 100 % Fe(II)).

**Supplementary figure S4.**


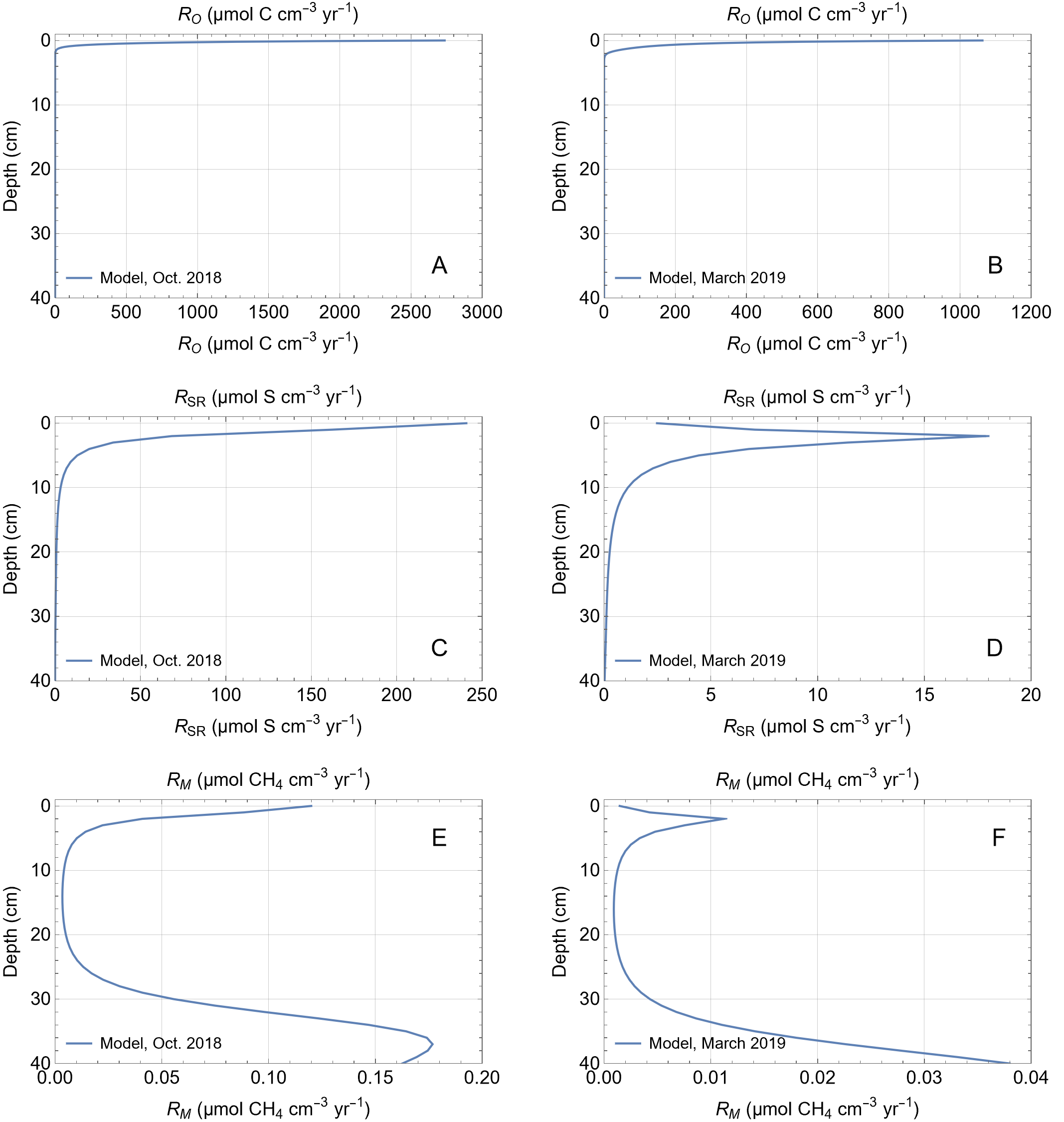


**Rates of particulate organic matter (POC) degradation calculated in the model for October 2018 and March 2019.** A and B: POC degradation rate in oxic–suboxic surface layer, C and D: Rate of dissimilatory sulfate reduction, E and F: Rate of methanogenesis.

**Supplementary figure S5.**


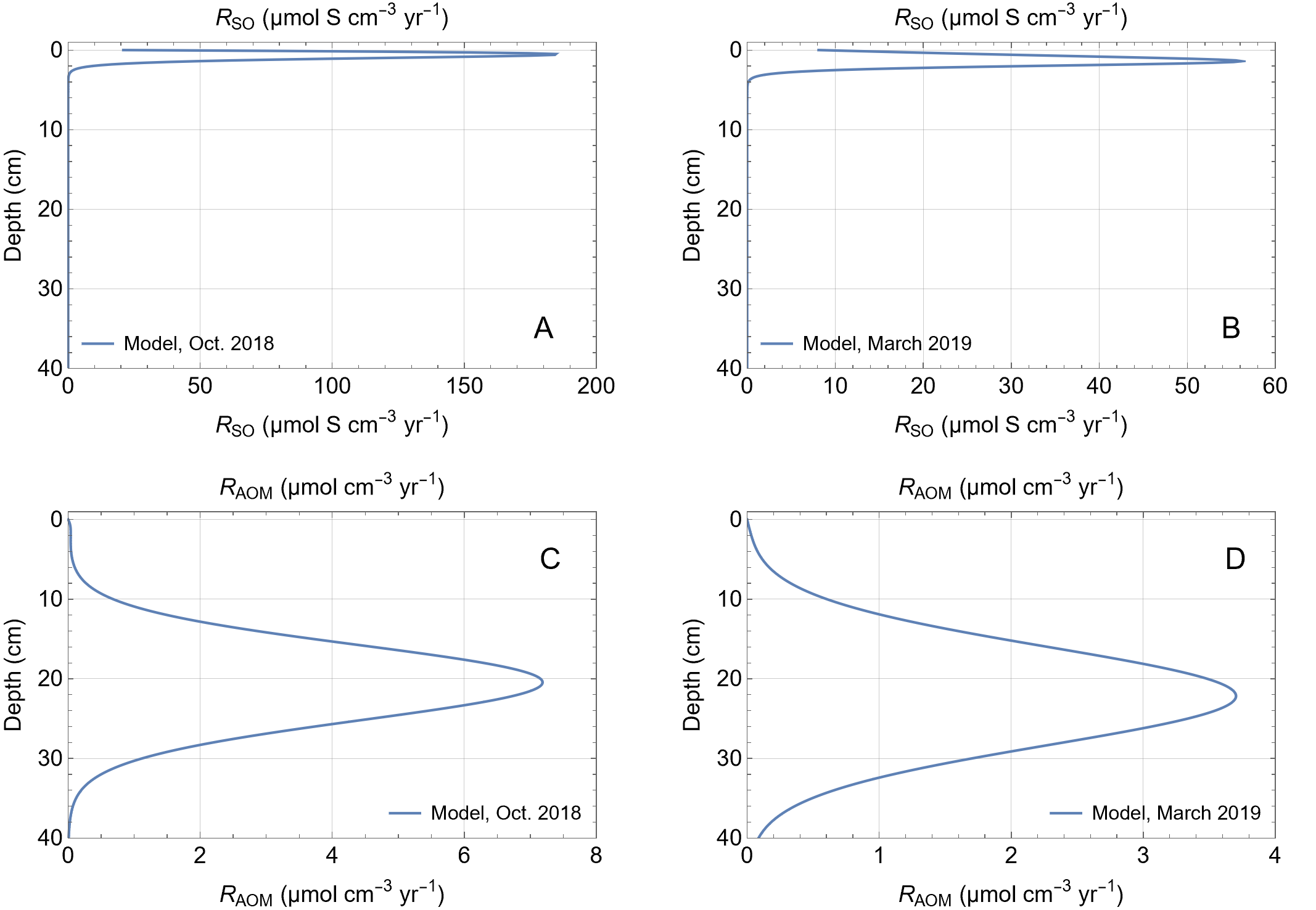


**Rates of secondary redox reactions calculated in the model for October 2018 and March 2019.** A and B: Rate of sulfide oxidation in oxic–suboxic surface layer, C and D: Rate of anaerobic oxidation of methane.

**Supplementary figure S6.**


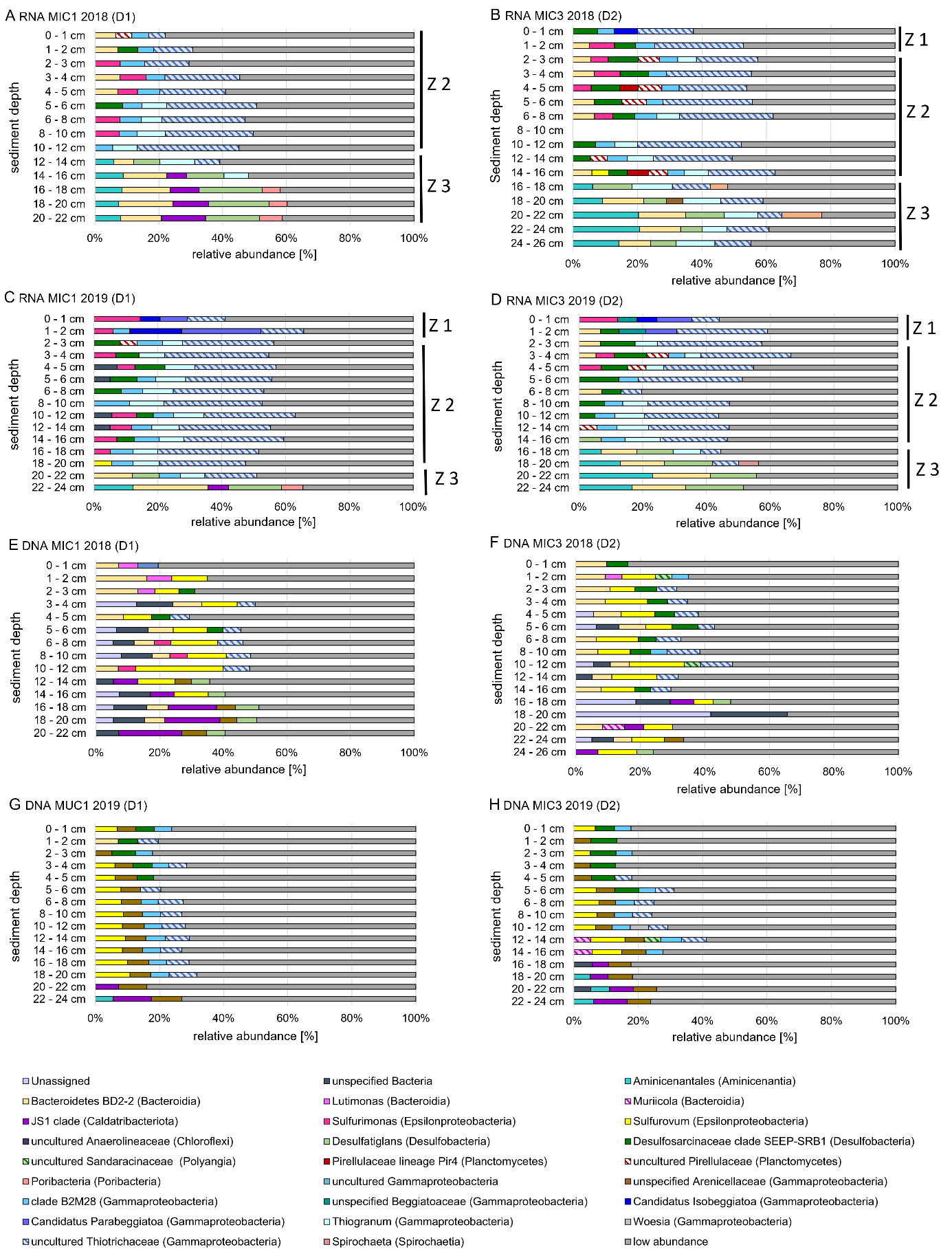


**Taxonomy plots of bacterial 16S tags** (>3%) for RNA (A-D) and DNA (E-H) for October 2018 and March 2019. Z=Zone

**Supplementary figure S7.**

**
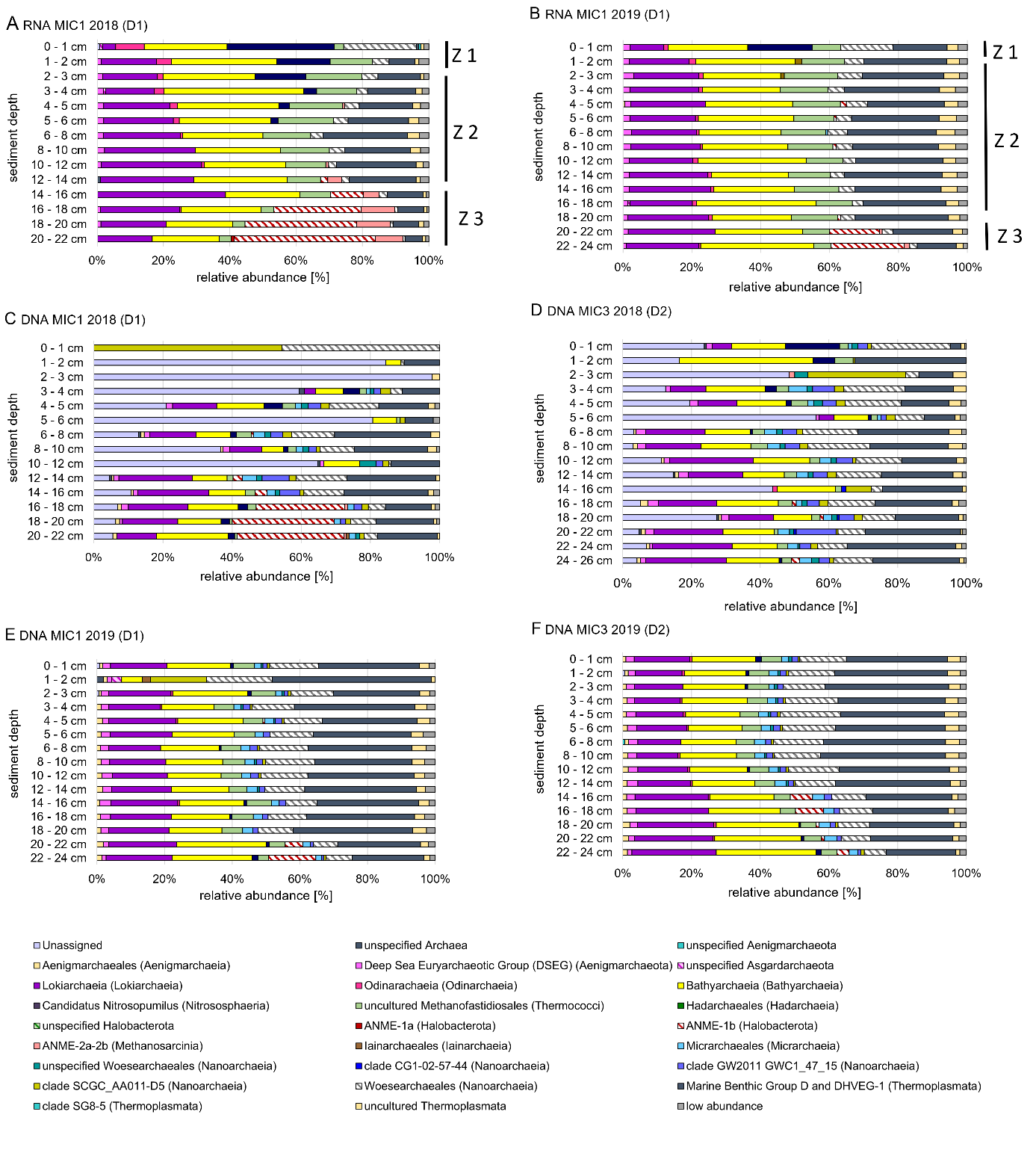
**

**Taxonomy plots of archaeal 16S tags** for RNA (A and B) and DNA (C-F) for October 2018 and March 2019. Z=Zone

**Supplementary Figure S8**


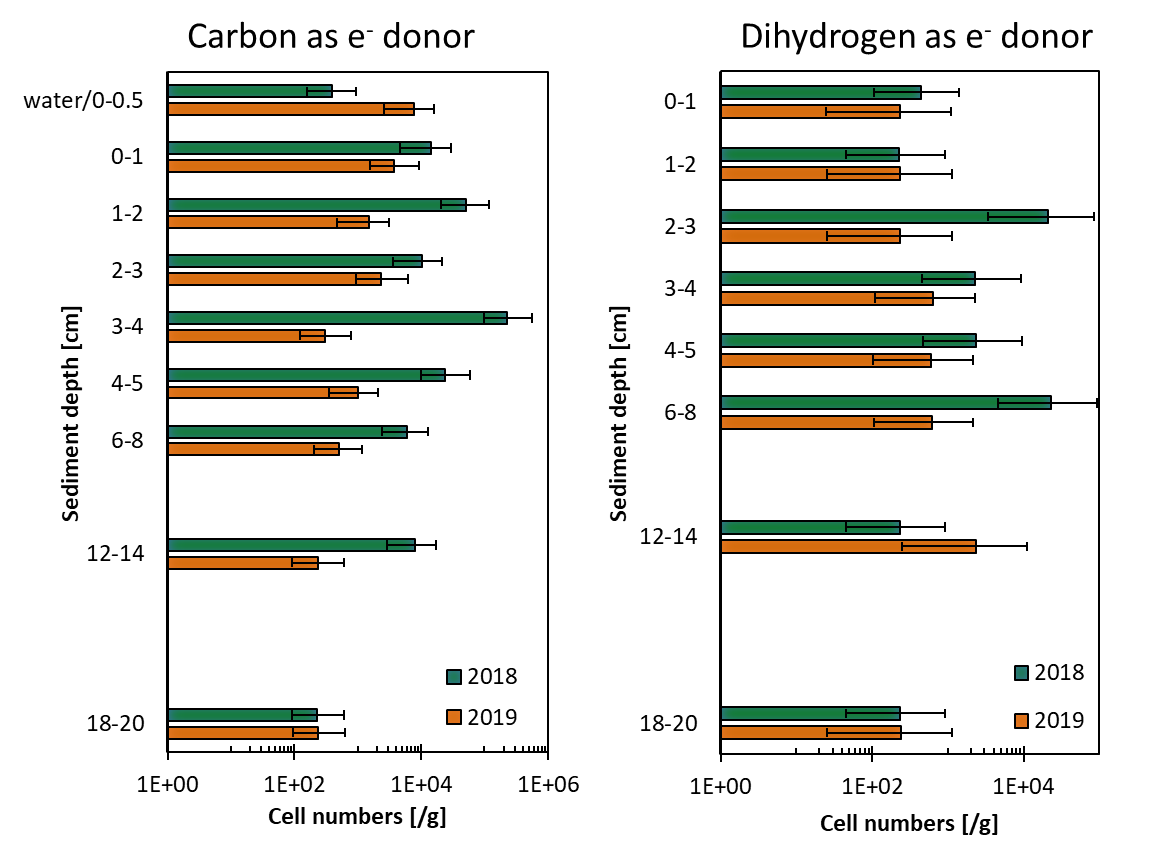


**Most probable number estimate of Fe(III)-reducing bacteria using acetate/lactate (left) or dihydrogen (right) as electron donor.**

**Supplementary figure S9.**

**
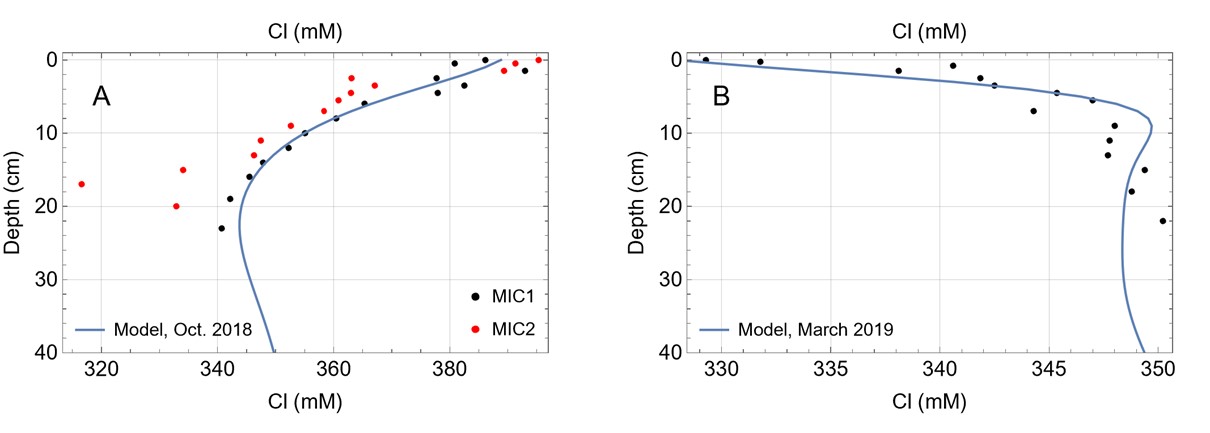
**

**Model results and data for dissolved chloride in sediment porewater from October 23^rd^ 2018 (A) and March 15^th^ 2019 (B).**

**Supplementary figure S10.**


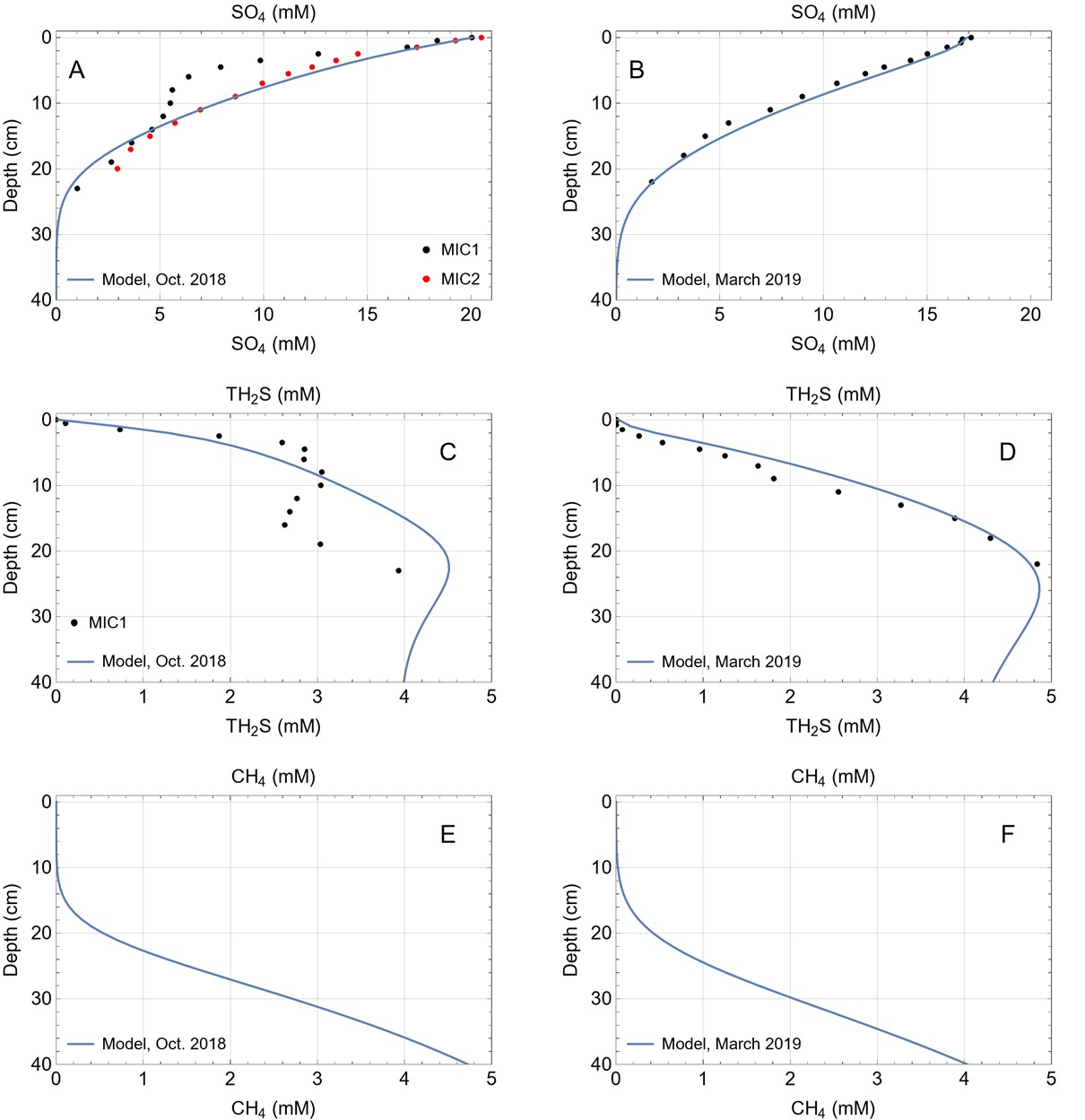


**Model results and data for dissolved sulfate, sulfide and methane at October 23^th^ 2018 and March 15^th^ 2019.** A and B: dissolved sulfate, C and D: dissolved sulfide, E and F: dissolved methane (no methane data available).

**Figure S11**


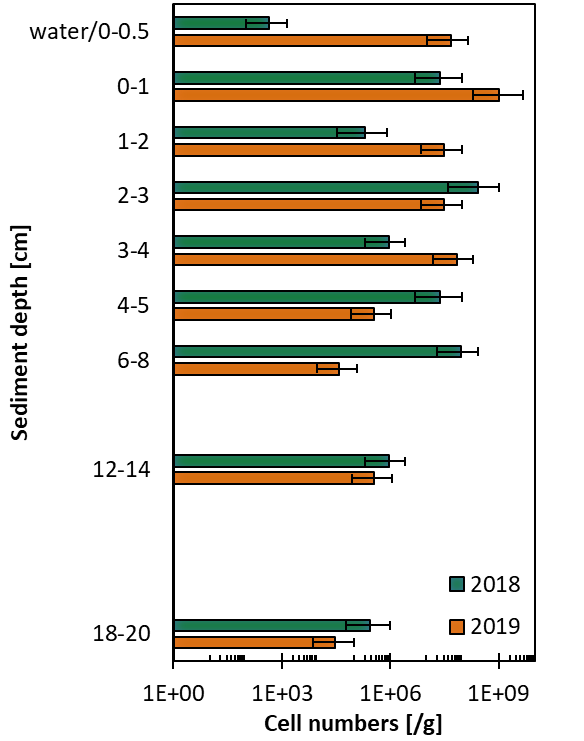


**Most probable number estimates of cells which grow under conditions conducive to microaerophilic Fe(II)-oxidizing conditions.** 2018 estimates come from incubations in ZVI plates whilst 2019 estimates come from growth in gradient tubes.

**Figure S12**


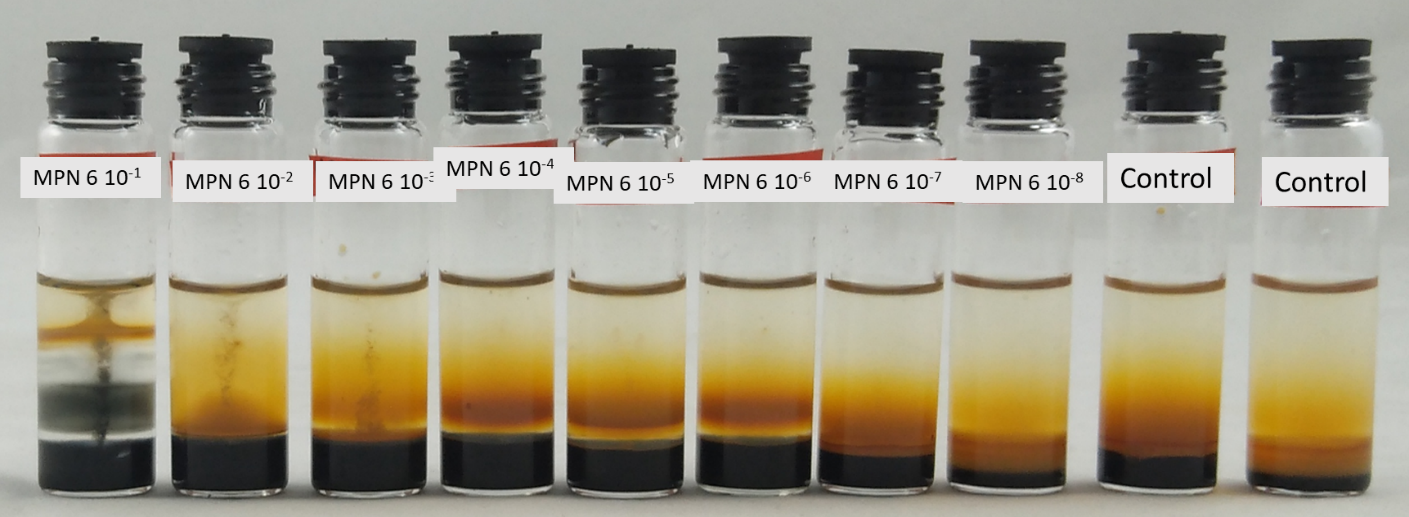
**An example dilution series in gradient tubes. Growth of microaerophilic Fe(II)-oxidizers shown by orange bands.** Black precipitates at low dilutions likely indicative of subsequent reduction of oxides formed in the tubes.

**Supplementary figure S13.**


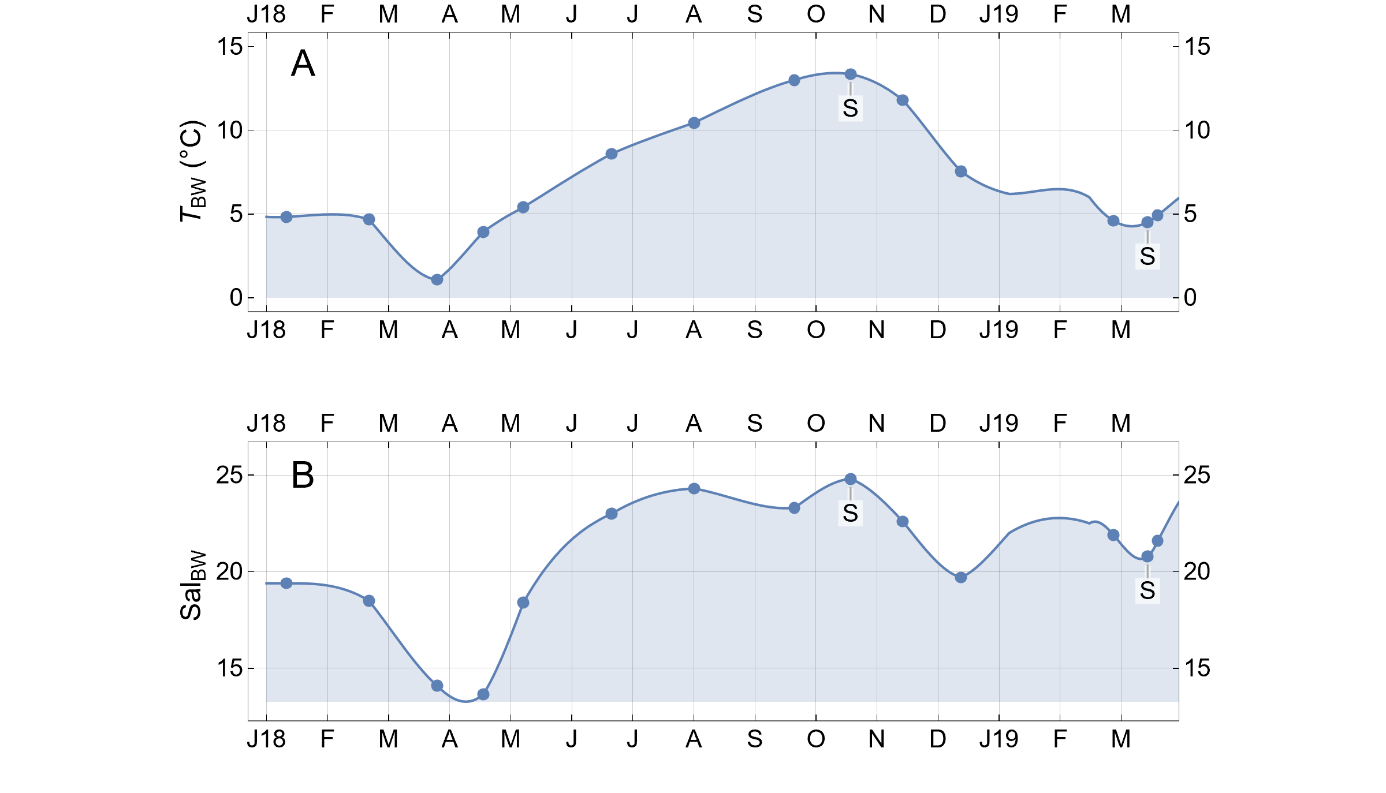


**Bottom water temperature (A) and salinity (B) applied as time-dependent upper boundary condition.** Symbols indicate data measured at 25 m water depth, lines are interpolation functions fitted through the data that are employed as upper boundary condition. The modeling period extends from Jan. 1^st^, 2018 to March 31^st^ 2019. Sediment cores were taken in Oct. 2018 and March 2019. Sampling dates are marked by “S”.

**Supplementary figure S14.**

**
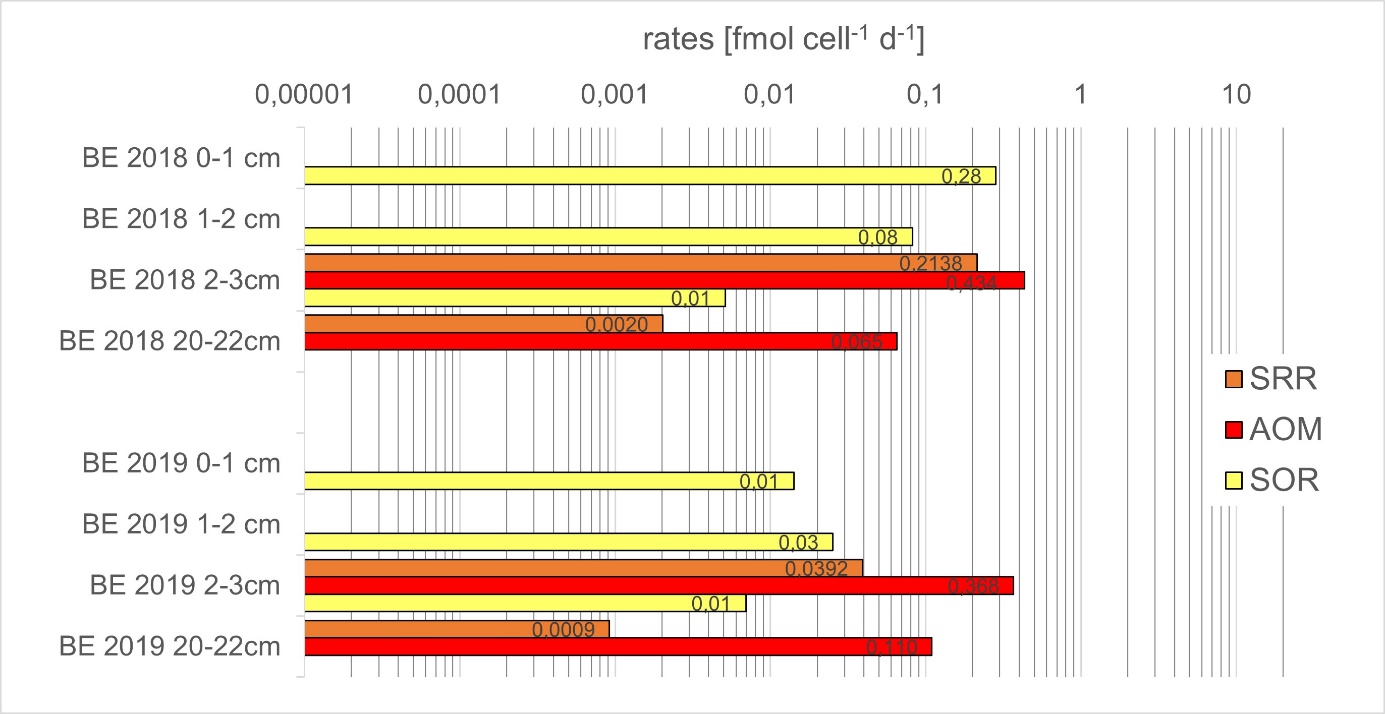
**

**Estimated per cell AOM, SR and SO rates for sediments from October 2018 and March 2019.**

**Supplementary figure S15.**

**
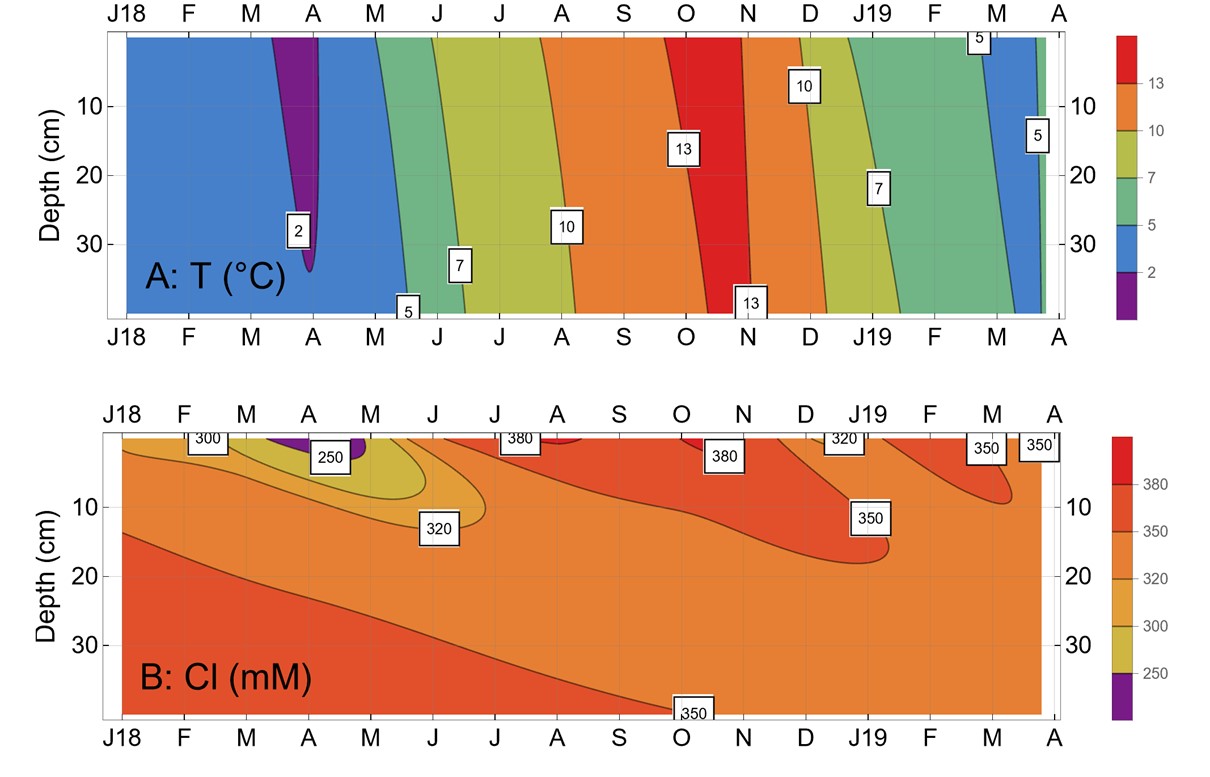
**

**Model results for temperature (A) and dissolved chloride (B) in the sediments.**

**Supplementary figure S16.**


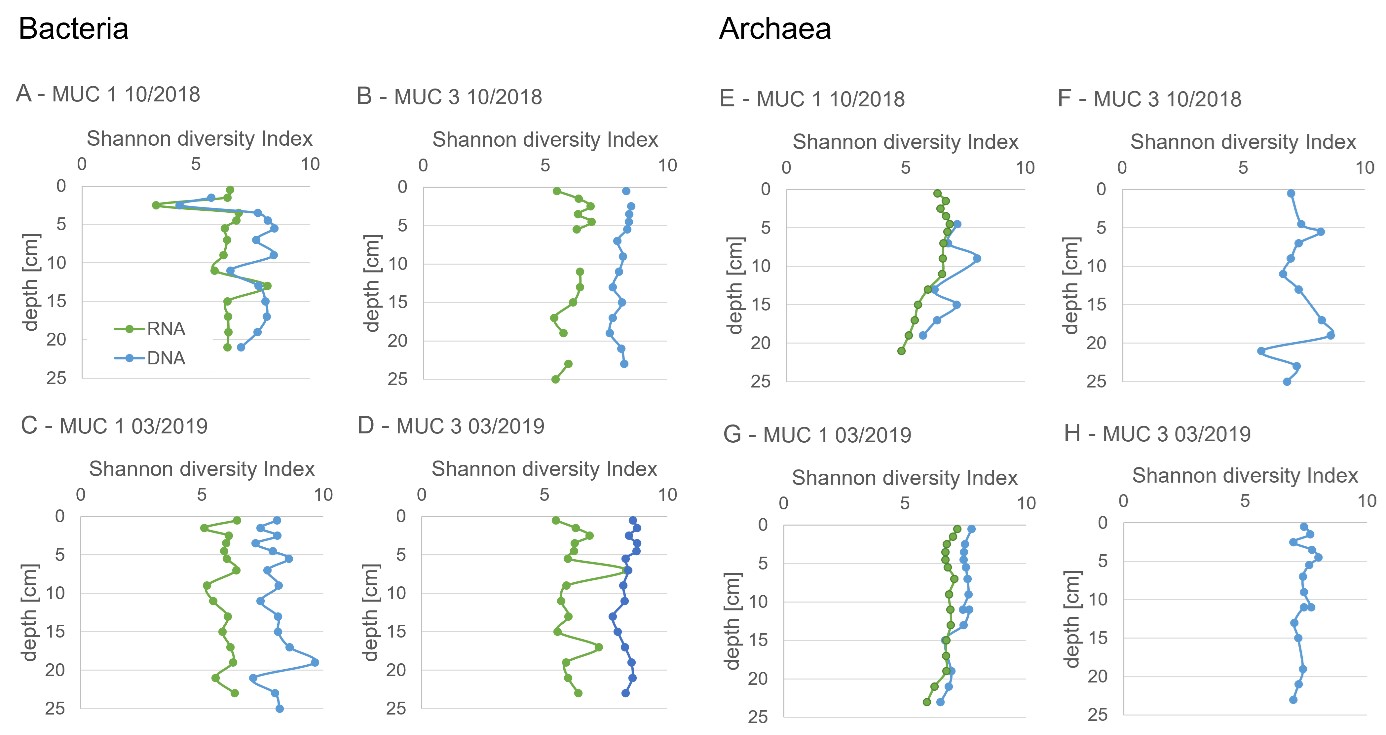


**Shannon diversity indices for DNA- and RNA-based analyses of Bacteria and Archaea in the different sediment cores.**

**Supplementary figure S17.**


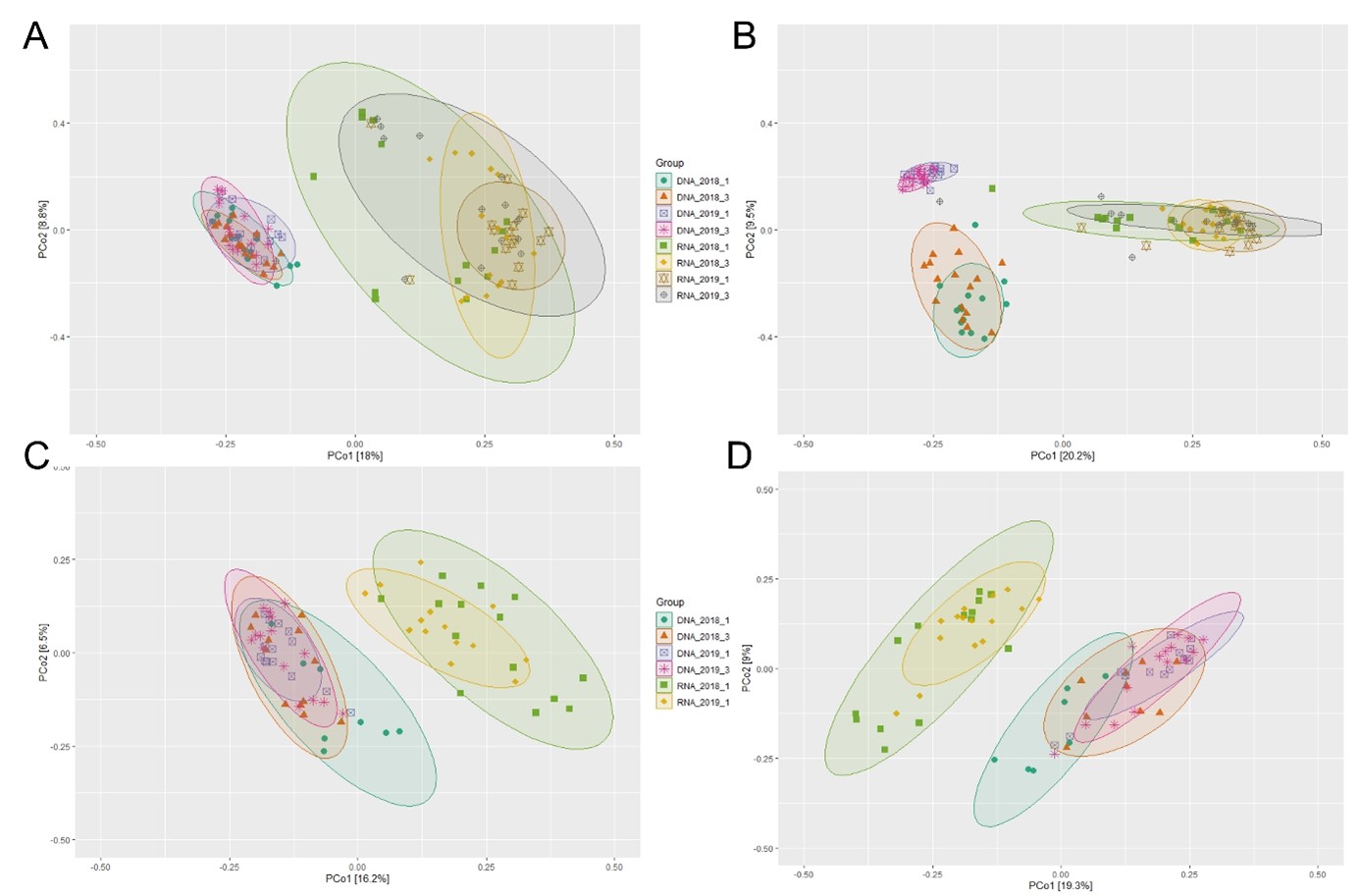


**Unweighted Principal coordinate analyses (PCoA) for RNA from Bacteria (A, B) and Archaea (C, D).** The threshold of sampling depth is set for 1000 sequences (A, C) and 2053 for bacterial (B) and 2625 for archaeal (D) sequences. The first two principal coordinates (PCo1 and PCo2) are plotted. Similarities and dissimilarities of the microbial communities are visualized for sediments collected in October 2018 (MIC1 and MIC3) and March 2019 (MIC1 and MIC3).

**References**

Abegg, F., and Anderson, A.L. (1997). The acoustic turbid layer in muddy sediments of Eckernförde Bay, Western Baltic: Methane concentration, saturation and bubble characteristics. *Mar. Geol.* 137**,** 137–147.

Adam, N., Kriete, C., Garbe-Schönberg, D., Gonnella, G., Krause, S., Schippers, A., et al. (2019). Microbial Community Compositions and Geochemistry of Sediments with Increasing Distance to the Hydrothermal Vent Outlet in the Kairei Field. *Geomicrobiol. J.* 37**,** 242-254.

Bange, H.W., Bergmann, K., Hansen, H.P., Kock, A., Koppe, R., Malien, F., et al. (2010). Dissolved methane during hypoxic events at the Boknis Eck time series station (Eckernförde Bay, SW Baltic Sea). *Biogeosciences* 7**,** 1279-1284.

Blazewicz, S.J., Barnard, R.L., Daly, R.A., and Firestone, M.K. (2013). Evaluating rRNA as an indicator of microbial activity in environmental communities: limitations and uses. *ISME J.* 7**,** 2061-2068.

Bokulich, N.A., Kaehler, B.D., Rideout, J.R., Dillon, M., Bolyen, E., Knight, R., et al. (2018). Optimizing taxonomic classification of marker-gene amplicon sequences with QIIME 2’s q2-feature-classifier plugin. *Microbiome* 6**,** 90.

Bolyen, E., Rideout, J.R., Dillon, M.R., Bokulich, N.A., Abnet, C.C., Al-Ghalith, G.A., et al. (2019). Reproducible, interactive, scalable and extensible microbiome data science using QIIME 2. *Nat. Biotechnol.* 37**,** 852-857.

Boudreau, B.P. (1997). *Diagenetic Models and Their Implementation.* Berlin: Springer-Verlag.

Callahan, B.J., Mcmurdie, P.J., Rosen, M.J., Han, A.W., Johnson, A.J.A., and Holmes, S.P. (2016). DADA2: High-resolution sample inference from Illumina amplicon data. *Nat. Methods* 13**,** 581-583.

Canfield, D.E., Raiswell, R., Westrich, J.T., Reaves, C.M., and Berner, R.A. (1986). The use of chromium reduction in the analysis of reduced inorganic sulfur in sediments and shales. *Chem. Geol.* 54**,** 149-155.

Cerqueira, T., Pinho, D., Froufe, H., Santos, R.S., Bettencourt, R., and Egas, C. (2017). Sediment Microbial Diversity of Three Deep-Sea Hydrothermal Vents Southwest of the Azores. *Microb. Ecol.* 74**,** 332-349.

Cussler, E.L. (1984). *Diffusion - Mass Transfer in Fluid Systems.* Cambridge: Cambridge University Press.

Dale, A.W., Bertics, V.J., Treude, T., Sommer, S., and Wallmann, K. (2013). Modeling benthic-pelagic nutrient exchange processes and porewater distributions in a seasonally hypoxic sediment: evidence for massive phosphate release by *Beggiatoa*? *Biogeosciences* 10**,** 629-651.

De Cena, J.A., Zhang, J., Deng, D., Dame-Teixeira, N., and Do, T. (2021). Low-Abundant Microorganisms: The Human Microbiome's Dark Matter, a Scoping Review. *Front. Cell Infect. Microbiol.* 11**,** 689197.

De Vrieze, J., Pinto, A.J., Sloan, W.T., and Ijaz, U.Z. (2018). The active microbial community more accurately reflects the anaerobic digestion process: 16S rRNA (gene) sequencing as a predictive tool. *Microbiome* 6**,** 63.

Ding, J., Zhang, Y., Wang, H., Jian, H., Leng, H., and Xiao, X. (2017). Microbial Community Structure of Deep-sea Hydrothermal Vents on the Ultraslow Spreading Southwest Indian Ridge. *Front Microbiol* 8**,** 1012.

Emerson, D., and Moyer, C. (1997). Isolation and characterization of novel iron-oxidizing bacteria that grow at circumneutral pH. *Appl. Environ. Microbiol.* 63**,** 4784-4792.

Evans, P.N., Parks, D.H., Chadwick, G.L., Robbins, S.J., Orphan, V.J., Golding, S.D., et al. (2015). Methane metabolism in the archaeal phylum Bathyarchaeota revealed by genome-centric metagenomics. *Science* 350**,** 434-438.

Garcia, J.-L., Patel, B.K.C., and Ollivier, B. (2000). Taxonomic, Phylogenetic, and Ecological Diversity of Methanogenic Archaea. *Anaerobe* 6**,** 205-226.

Gaye, B., Lahajnar, N., Harms, N., Paul, S.a.L., Rixen, T., and Emeis, K.C. (2022). What can we learn from amino acids about oceanic organic matter cycling and degradation? *Biogeosciences* 19**,** 807-830.

Gindorf, S., Bange, H.W., Booge, D., and Kock, A. (2022). Seasonal study of the small-scale variability of dissolved methane in the western Kiel Bight and Eckernförde Bay (Baltic Sea) during the European heat wave in 2018. *Biogeosciences* 19**,** 4993-5006.

Grasshoff, K., Kremling, K., and Ehrhardt, M. (1999). *Methods of seawater analysis.* New York, : Wiley-VCH.

Huang, W.C., Liu, Y., Zhang, X., Zhang, C.J., Zou, D., and Zheng, S. (2021). Comparative genomic analysis reveals metabolic flexibility of Woesearchaeota. *Nat. Commun.* 12**,** 5281.

Ishii, K., Mussmann, M., Macgregor, B.J., and Amann, R. (2004). An improved fluorescence in situ hybridization protocol for the identification of bacteria and archaea in marine sediments. *FEMS Microbiol. Ecol.* 50**,** 203-213.

Ivanenkov, V.N., and Lyakhin, Y.I. (1978). "Determination of total alkalinity in seawater.," in *Methodes of hydrochemical investigations in the ocean,* eds. O.K. Bordovsky & V.N. Ivanenkov. (Moscow: Nauka Publ. House,), 110-114.

Jochum, L.M., Chen, X., Lever, M.A., Loy, A., Jorgensen, B.B., Schramm, A., et al. (2017). Depth Distribution and Assembly of Sulfate-Reducing Microbial Communities in Marine Sediments of Aarhus Bay. *Appl. Environ. Microbiol.* 83**,** <https://doi.org/10.1128/AEM.01547-01517>.

Jochum, L.M., Schreiber, L., Marshall, I.P.G., Jorgensen, B.B., Schramm, A., and Kjeldsen, K.U. (2018). Single-Cell Genomics Reveals a Diverse Metabolic Potential of Uncultivated *Desulfatiglans*-Related Deltaproteobacteria Widely Distributed in Marine Sediment. *Front. Microbiol.* 9**,** 2038.

Jørgensen, B.B. (1977). Distribution of colorless sulfur bacteria (*Beggiatoa* spp.) in a coastal marine sediment. *Mar. Biol.* 41**,** 19-28.

Kadnikov, V., Mardanov, A., Beletsky, A., Karnachuk, O., and Ravin, N. (2019 ). Genome of the candidate phylum Aminicenantes bacterium from a deep subsurface thermal aquifer revealed its fermentative saccharolytic lifestyle. *Extremophiles* 23**,** 189-200.

Kamke, J., Sczyrba, A., Ivanova, N., Schwientek, P., Rinke, C., Mavromatis, K., et al. (2013). Single-cell genomics reveals complex carbohydrate degradation patterns in poribacterial symbionts of marine sponges. *ISME J.* 7**,** 2287-2300.

Kerou, M., Ponce-Toledo, R.I., Zhao, R., Abby, S.S., Hirai, M., Nomaki, H., et al. (2021). Genomes of Thaumarchaeota from deep sea sediments reveal specific adaptations of three independently evolved lineages. *ISME J.* 15**,** 2792-2808.

Kevorkian, R.T., Callahan, S., Winstead, R., and Lloyd, K.G. (2021). ANME-1 archaea may drive methane accumulation and removal in estuarine sediments. *Environ. Microbiol. Rep.* 13**,** 185-194.

Klee, A.J. (1993). A computer program for the determination of most probable number and its confidence limits. *J. Microbiol. Methods* 18**,** 91-98.

Klindworth, A., Pruesse, E., Schweer, T., Peplies, J., Quast, C., Horn, M., et al. (2013). Evaluation of general 16S ribosomal RNA gene PCR primers for classical and next-generation sequencing-based diversity studies. *Nucleic Acids Res.* 41**,** e1.

Kostka, J.E., and Luther, G.W. (1994). Partitioning and Speciation of Solid-Phase Iron in Salt-Marsh Sediments. *Geochim. Cosmochim. Acta* 58**,** 1701-1710.

Kueh, M.-T., and Lin, C.-Y. (2020). The 2018 summer heatwaves over northwestern Europe and its extended-range prediction. *Sci Rep-Uk* 10.

Lapoussière, A., Michel, C., Gosselin, M., Poulin, M., Martin, J., and Tremblay, J.-É. (2013). Primary production and sinking export during fall in the Hudson Bay system, Canada. *Cont. Shelf Res.* 52**,** 62-72.

Laufer, K., Nordhoff, M., Røy, H., Schmidt, C., Behrens, S., Jørgensen, B.B., et al. (2016). Coexistence of microaerophilic, nitrate-reducing, and phototrophic Fe (II) oxidizers and Fe (III) reducers in coastal marine sediment. *Appl. Environ. Microbiol.* 82**,** 1433-1447.

Lee, Y.M., Hwang, K., Lee, J.I., Kim, M., Hwang, C.Y., Noh, H.J., et al. (2018). Genomic Insight Into the Predominance of Candidate Phylum Atribacteria JS1 Lineage in Marine Sediments. *Front Microbiol* 9**,** 2909.

Lennartz, S.T., Lehmann, A., Herrford, J., Malien, F., Hansen, H.P., Biester, H., et al. (2014). Long-term trends at the Boknis Eck time series station (Baltic Sea), 1957-2013: does climate change counteract the decline in eutrophication? *Biogeosciences* 11**,** 6323-6339.

Lewandowska, A.M., Boyce, D.G., Hofmann, M., Matthiessen, B., Sommer, U., and Worm, B. (2014). Effects of sea surface warming on marine plankton. *Ecol. Lett.* 17**,** 614-623.

Li, M., Yang, Y., Mi, T., He, H., and Zhen, Y. (2020). Differences between DNA- and RNA-Based Bacterial Communities in Marine Sediments. *Huan jing ke xue= Huanjing kexue / [bian ji, Zhongguo ke xue yuan huan jing ke xue wei yuan hui "Huan jing ke xue" bian ji wei yuan hui.]* 41**,** 2485-2495.

Liu, C., Cui, Y., Li, X., and Yao, M. (2021). microeco: an R package for data mining in microbial community ecology. *FEMS Microbiol. Ecol.* 26**,** fiaa255.

Lloyd, K.G., Steen, A.D., Ladau, J., Yin, J., and Crosby, L. (2018). Phylogenetically Novel Uncultured Microbial Cells Dominate Earth Microbiomes. *Msystems* 3.

Lomas, M.W., Bates, N.R., Johnson, R.J., Steinberg, D.K., and Tanioka, T. (2022). Adaptive carbon export response to warming in the Sargasso Sea. *Nat. Commun.* 13**,** 1211.

Ma, X., Sun, M., Lennartz, S.T., and Bange, H.W. (2020). A decade of methane measurements at the Boknis Eck Time Series Station in Eckernförde Bay (southwestern Baltic Sea). *Biogeosciences* 17**,** 3427-3438.

Maltby, J., Steinle, L., Loscher, C.R., Bange, H.W., Fischer, M.A., Schmidt, M., et al. (2018). Microbial methanogenesis in the sulfate-reducing zone of sediments in the Eckernforde Bay, SW Baltic Sea. *Biogeosciences* 15**,** 137-157.

Middelburg, J.J. (1989). A simple model for organic matter decomposition in marine sediments. *Geochim. Cosmochim. Acta* 53**,** 1577-1581.

Millero, F.J. (1996). *Chemical Oceanography.* Boca Raton: CRC Press.

Nobu, M.K., Dodsworth, J.A., Murugapiran, S.K., Rinke, C., Gies, E.A., Webster, G., et al. (2016). Phylogeny and physiology of candidate phylum ‘Atribacteria’ (OP9/JS1) inferred from cultivation-independent genomics. *ISME J.* 10**,** 273-286.

Orcutt, B., and Meile, C. (2008). Constraints on mechanisms and rates of anaerobic oxidation of methane by microbial consortia: process-based modeling of ANME-2 archaea and sulfate reducing bacteria interactions. *Biogeosci. Disc.* 5**,** 1587–1599.

Oremland, R.S., and Polcin, S. (1982). Methanogenesis and sulfate reduction: competitive and noncompetitive substrates in estuarine sediments. *Appl. Environ. Microbiol.* 44**,** 1270–1276.

Orsi, W.D., Vuillemin, A., Rodriguez, P., Coskun, Ö.K., Gomez-Saez, G.V., Lavik, G., et al. (2020). Metabolic activity analyses demonstrate that Lokiarchaeon exhibits homoacetogenesis in sulfidic marine sediments. *Nat. Microbiol.* 5**,** 248-255.

Otte, J.M., Harter, J., Laufer, K., Blackwell, N., Straub, D., Kappler, A., et al. (2018). The distribution of active iron-cycling bacteria in marine and freshwater sediments is decoupled from geochemical gradients. *Environ. Microbiol.* 20**,** 2483-2499.

Pedregosa, F., Varoquaux, G., Gramfort, A., Michel, V., Thirion, B., Grisel, O., et al. (2011). Scikit-learn: Machine Learning in Python. *J. Mach. Learn. Res.* 12**,** 2825-2830.

Pernthaler, A., Pernthaler, J., and Amann, R. (2002). Fluorescence in situ hybridization and catalyzed reporter deposition for the identification of marine bacteria. *Appl. Environ. Microbiol.* 68**,** 3094-3101.

Poulsen, M., Schwab, C., Borg Jensen, B., Engberg, R.M., Spang, A., Canibe, N., et al. (2013). Methylotrophic methanogenic Thermoplasmata implicated in reduced methane emissions from bovine rumen. *Nat. Commun.* 4**,** 1428.

Preisler, A., De Beer, D., Lichtschlag, A., Lavik, G., Boetius, A., and Jørgensen, B.B. (2007). Biological and chemical sulfide oxidation in a Beggiatoa inhabited marine sediment. *ISME J.* 1**,** 341-353.

Price, M.N., Dehal, P.S., and Arkin, A.P. (2010). FastTree 2-approximately maximum-likelihood trees for large alignments. *PloS one* 5**,** e9490.

Quast, C., Pruesse, E., Yilmaz, P., Gerken, J., Schweer, T., Yarza, P., et al. (2013). The SILVA ribosomal RNA gene database project: improved data processing and web-based tools. *Nucleic Acids Res.* 41**,** D590-596.

Raven, K.P., Jain, A., and Loeppert, R.H. (1998). Arsenite and arsenate adsorption on ferrihydrite: kinetics, equilibrium, and adsorption envelopes. *Environ. Sci. Technol.* 32**,** 344-349.

Schippers, A., Kock, D., Hoft, C., Koweker, G., and Siegert, M. (2012). Quantification of Microbial Communities in Subsurface Marine Sediments of the Black Sea and off Namibia. *Front. Microbiol.* 3**,** 16.

Schwertmann, U., and Cornell, R.M. (2008). *Iron oxides in the laboratory: preparation and characterization.* John Wiley & Sons.

Sela-Adler, M., Ronen, Z., Herut, B., Antler, G., Vigderovich, H., Eckert, W., et al. (2017). Co-existence of Methanogenesis and Sulfate Reduction with Common Substrates in Sulfate-Rich Estuarine Sediments. *Front. Microbiol.* 8**,** <https://doi.org/10.3389/fmicb.2017.00766>.

Spang, A., Saw, J.H., Jørgensen, S.L., Zaremba-Niedzwiedzka, K., Martijn, J., Lind, A.E., et al. (2015). Complex archaea that bridge the gap between prokaryotes and eukaryotes. *Nature* 521**,** 173-179.

Steinle, L., Maltby, J., Treude, T., Kock, A., Bange, H.W., Engbersen, N., et al. (2017). Effects of low oxygen concentrations on aerobic methane oxidation in seasonally hypoxic coastal waters. *Biogeosciences* 14**,** 1631-1645.

Stolpovsky, K., Dale, A.W., and Wallmann, K. (2015). Toward a parameterization of global-scale organic carbon mineralization kinetics in surface marine sediments. *Global Biogeochem Cy* 29**,** 812-829.

Stookey, L.L. (1970). Ferrozine - a new spectrophotometric reagent for iron. *Anal. Chem.* 42**,** 779-781.

Suzuki, D., Li, Z., Cui, X., Zhang, C., and Katayama, A. (2014). Reclassification of *Desulfobacterium anilini* as *Desulfatiglans anilini* comb. nov. within *Desulfatiglans* gen. nov., and description of a 4-chlorophenol-degrading sulfate-reducing bacterium, *Desulfatiglans parachlorophenolica* sp. nov. *Int. J. Syst. Evol. Microbiol.* 64**,** 3081-3086.

Torti, A., Lever, M.A., and Jørgensen, B.B. (2015). Origin, dynamics, and implications of extracellular DNA pools in marine sediments. *Mar. Genom.* 24**,** 185-196.

Treude, T., Kruger, M., Boetius, A., and Jorgensen, B.B. (2005). Environmental control on anaerobic oxidation of methane in the gassy sediments of Eckernforde Bay (German Baltic). *Limnol. Oceanogr.* 50**,** 1771-1786.

Tschech, A., and Pfennig, N. (1984). Growth yield increase linked to caffeate reduction in *Acetobacterium woodii*. *Arch. Microbiol.* 137**,** 163-167.

Von Hoyningen-Huene, A.J.E., Schneider, D., Fussmann, D., Reimer, A., Arp, G., and Daniel, R. (2022). DNA- and RNA-based bacterial communities and geochemical zonation under changing sediment porewater dynamics on the Aldabra Atoll. *Sci Rep-Uk* 12**,** 4257.

Widdel, F. (1980). *Anaerober Abbau von Fettsäuren und Benzoesäure durch neu isolierte Arten Sufat reduzierender Bakterien.* PhD PhD thesis, Göttingen University.

Widdel, F., and Pfennig, N. (1981). Studies on dissimilatory sulfate-reducing bacteria that compose fatty acids. *Arch. Microbiol.* 129**,** 395–400.

Winogradsky, S. (1887). Über Schwefelbakterien. *Botanische Zeitung* 45**,** 489-610.

Zamkovaya, T., Foster, J.S., De Crécy-Lagard, V., and Conesa, A. (2021). A network approach to elucidate and prioritize microbial dark matter in microbial communities. *ISME J.* 15**,** 228-244.

Zhang, C.-J., Pan, J., Liu, Y., Duan, C.-H., and Li, M. (2020). Genomic and transcriptomic insights into methanogenesis potential of novel methanogens from mangrove sediments. *Microbiome* 8**,** 94.

Zhou, Z., Liu, Y., Lloyd, K.G., Pan, J., Yang, Y., Gu, J.-D., et al. (2019). Genomic and transcriptomic insights into the ecology and metabolism of benthic archaeal cosmopolitan, Thermoprofundales (MBG-D archaea). *ISME J.* 13**,** 885-901.

Zhou, Z., Pan, J., Wang, F., Gu, J.-D., and Li, M. (2018). Bathyarchaeota: globally distributed metabolic generalists in anoxic environments. *FEMS Microbiol. Rev.* 42**,** 639-655.
